# Effects of Omega‐3 Fatty Acids Intake on Lipid Metabolism and Plaque Volume in Patients With Coronary Heart Disease: A Systematic Review and Dose–Response Meta‐Analysis of Randomized Clinical Trials

**DOI:** 10.1002/fsn3.70372

**Published:** 2025-06-02

**Authors:** Chunyu Zhang, Yulei Xie, Jie Zhou, Chun Zhang, Qilang Xiang, Yi Zhong, Juan Xiao, Jian Feng, Bin Liao, Xuxin Chen, Li Deng

**Affiliations:** ^1^ Department of Cardiology The Affiliated Hospital of Southwest Medical University, Immunity and Regeneration Key Laboratory of Luzhou Luzhou Sichuan China; ^2^ Department of Rehabilitation Medicine The Affiliated Hospital of North Sichuan Medical College Nanchong Sichuan China; ^3^ School of Rehabilitation Capital Medical University Beijing China; ^4^ Department of Dermatology The Affiliated Hospital of Southwest Medical University Luzhou Sichuan China; ^5^ Department of Cardiology Hechuan District People's Hospital Chongqing China; ^6^ Department of Gastroenterology The Affiliated Hospital of Southwest Medical University Luzhou China; ^7^ Department of Cardiovascular Surgery The Affiliated Hospital of Southwest Medical University, Metabolic Vascular Diseases Key Laboratory of Sichuan Province Luzhou Sichuan China; ^8^ Department of Pulmonary and Critical Care Medicine, the Sixth Medical Center of Chinese PLA General Hospital Beijing China; ^9^ Department of Rheumatology The Affiliated Hospital of Southwest Medical University Luzhou Sichuan China

**Keywords:** arterial plaque, blood lipids, coronary heart disease, Omega‐3 fatty acids

## Abstract

This systematic review and meta‐analysis of 23 randomized controlled trials (RCTs) encompassed an overall sample size of 2061 patients diagnosed with coronary heart disease. The objective was to assess the impact of omega‐3 fatty acids intake on lipid profiles and arterial plaque volume. Omega‐3 fatty acids supplementation significantly reduced circulating levels of triglycerides (TGs) (standardized mean difference [SMD] = −0.25, 95% confidence interval [CI] = −0.38 to −0.11) and total cholesterol (TC) (SMD = −0.12, 95% CI = −0.23 to −0.02), with no significant impact on the levels of high‐density lipoprotein cholesterol (HDL‐C), low‐density lipoprotein cholesterol (LDL‐C), or arterial plaque volume. Our omega‐3 fatty acids dose–response analysis revealed a linear relationship with TG and a “J” shaped curve for TC and LDL‐C levels. Our findings suggest that omega‐3 fatty acids intake had a positive impact on the circulating levels of TG and TC. Our findings underscore the importance of tailoring omega‐3 fatty acids dosage to individual patient needs in clinical practice.

**Trial Registration:** PROSPERO:CRD42023486434

## Graphical abstract

Omega‐3 fatty acids supplementation significantly reduced circulating levels of triglycerides (TG) (standardized mean difference [SMD] = −0.25, 95% confidence interval [CI] = −0.38 to −0.11) and total cholesterol (TC) (SMD = −0.12, 95% CI = −0.23 to −0.02), with no significant impact on the levels of high‐density lipoprotein cholesterol (HDL‐C), low‐density lipoprotein cholesterol (LDL‐C), or arterial plaque volume. Our omega‐3 fatty acids dose–response analysis revealed a linear relationship with TG and a “J” shaped curve for TC and LDL‐C levels. Our findings suggest that omega‐3 fatty acids intake had a positive impact on the circulating levels of TG and TC.

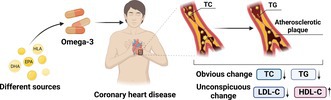

## 1 Introduction

With an increasingly aging population, coronary atherosclerotic heart disease has emerged as the leading cause of morbidity and mortality worldwide. Epidemiological investigations have shown that there are approximately 197 million individuals with coronary heart disease globally, resulting in over 9.1 million deaths per year (Safiri et al. 2022). A number of factors, including inflammation, endothelial dysfunction, dyslipidaemia, insulin resistance, environmental exposure, and pathogens, can influence the onset and progression of coronary atherosclerosis (Falk 2006), and dyslipidaemia is closely associated with it (Skålén et al. 2002; Bissell 2008; Catapano et al. 2016).

The category of omega‐3 fatty acids encompasses a variety of polyunsaturated fatty acids, notably eicosapentaenoic acid (EPA), docosahexaenoic acid (DHA), and alpha‐linolenic acid (ALA). These compounds serve as agents for managing and controlling blood lipids, along with statins, niacin, and fibrate drugs. However, adverse reactions to statins, niacin, and fibrates, such as abnormal liver function, muscle complications, hyperuricemia, gastrointestinal discomfort, and new‐onset diabetes, limit their application to specific demographic groups (Li et al. 2020; Lavigne and Karas 2013; Bays et al. 2014; Okopień et al. 2018). Omega‐3 fatty acids have received increasing attention in clinical practice owing to their relatively few side effects and good patient tolerability. Recently, several meta‐analyses examined the effects of omega‐3 fatty acids on lipid profiles and the presence of atherosclerotic plaques in the coronary arteries (Wang et al. 2023; Khan et al. 2021; Lee et al. 2023; Xiao et al. 2022; Mei et al. 2022; Gao et al. 2022). Wang et al. (2023) conducted a meta‐analysis revealing that omega‐3 fatty acid supplementation reduces triglyceride (TG) and nonhigh‐density lipoprotein cholesterol (HDL) levels in a dose‐dependent linear fashion. Nevertheless, including diverse populations comprising patients with CHD, hypercholesterolemia, and hypertriglyceridemia may have contributed to variability in the results. Likewise, a meta‐analysis by Gao et al. demonstrated that omega‐3 supplementation reduces the volume of atherosclerotic plaques (SMD −0.18; 95% CI −0.31 to −0.05) (Gao et al. 2022). However, the presence of data extraction errors in that analysis undermines the robustness of its findings. We are planning to conduct a new systematic review to evaluate the effects of omega‐3 fatty acid intake on lipid profiles and plaque volume in individuals with CHD. The findings will help clinicians optimize omega‐3 fatty acid intake strategies to better manage lipid profiles in patients with CHD.

This study focused on the following three aspects: (1) exploring the impact of omega‐3 fatty acids intake on TG, HDL‐C, LDL‐C, and TC levels, and coronary atherosclerotic plaques among individuals diagnosed with CHD. (2) Investigating the impact of different countries, mean ages, disease states, intervention durations, doses, types of omega‐3 fatty acids on lipid profiles through predefined subgroup analyses. (3) Employing a one‐stage three‐spline regression model (Crippa et al. 2019) to explore the dose–response relationship between omega‐3 fatty acids intake and TG, HDL‐C, LDL‐C, and TC levels, and coronary atherosclerotic plaques in patients with CHD.

## 2 Materials and Methods

The review process followed the checklist outlined in the PRISMA statement (Table S1) (Ardern et al. 2022). We registered our study at PROSPERO, with the identifier as CRD42023486434.

### 2.1 Search Strategies

The analysis encompassed randomized controlled trials (RCTs) exploring the consequences of omega‐3 fatty acid administration on lipid profiles in patients with coronary atherosclerotic disease, as documented in peer‐reviewed and English‐language journals. The articles were sourced from four databases—PubMed, Web of Science, Embase, and the Cochrane Library—up to March 30, 2025. The search strategy is outlined in detail in Table S2. The keywords used included “omega‐3 fatty acids,” “eicosapentaenoic acid (EPA),” “docosahexaenoic acid (DHA),” “alpha‐linolenic acid (ALA),” “coronary atherosclerotic heart disease,” “lipids,” and “plaque.” Moreover, we conducted a thorough examination of the bibliographies from prior systematic reviews and meta‐analyses to uncover additional relevant studies for potential inclusion in our research. Inter‐rater agreement was evaluated and reported using Cohen's kappa coefficient (κ) (Cohen 1960).

### 2.2 Inclusion and Exclusion Criteria

Our inclusion criteria were as follows: (1) the research employed a parallel RCT; (2) the intervention involved omega‐3 fatty acids including EPA or DHA or ALA (Siscovick et al. 2017); (3) participants were adults aged ≥ 18 years and were diagnosed with CAD by medical professionals; (4) the study included a control group (placebo or other comparators similar to omega‐3 fatty acids but without active ingredients); (5) the research documented one or more of these outcomes: circulating levels of TC and/or TG and/or LDL‐C and/or HDL‐C and/or coronary atherosclerotic plaque volume; (6) endpoint evaluations excluded cases of mortality; (7) only studies with complete follow‐up data were included to ensure that all data were available.

Our exclusion criteria were as follows: (1) cross‐sectional studies, reviews, preclinical studies, and any studies not relevant to the objectives; (2) studies with incomplete or noncompliant follow‐up procedures; (3) studies without outcome measures such as postintervention blood lipids or plaque; and (4) redundant reports.

### 2.3 Quality Evaluation

The revised Cochrane risk of bias tool (RoB2) was used to evaluate potential biases within RCTs (Sterne et al. 2019). Following the guidelines of the Cochrane Handbook, the risk of bias was classified as low, high, or unclear for various aspects that included: randomization, blinding, incomplete outcome data, measurement, selection of reporting outcomes, and others (Higgins et al. 2011). The risk of bias was independently evaluated for all reviewed studies by two investigators (Z.C.Y. and X.Y.L.). In cases of disagreement, a consensus was reached through collaborative dialogue with a third investigator (Z.C.) and, if necessary, through further consultation.

### 2.4 Data Extraction

Data extraction was conducted by two independent authors using the following criteria: authorship, publication year, location, study design, participant age and sex, number of participants in both intervention and control groups, type of intervention, follow‐up duration, and outcome measures. When reports were incomplete, we contacted the corresponding authors to seek additional data required for our analysis. In cases where trials assessed varying dosages of omega‐3 fatty acids, the results were analyzed as two distinct trials. Disagreements were addressed through discussions involving an additional member of the research team.

### 2.5 Statistical Analyses

All outcome variables extracted from the included studies were continuous and expressed as means and standard deviations (SD). The mean differences (MD) or standardized mean differences (SMDs) were calculated as measures of effect (Higgins et al. 2011), with 95% confidence intervals (CI). The values for mean change and SD change from baseline to end of intervention were used for dose–response analysis. These values were calculated using measurements taken before and after the intervention and the formula: SD^2^ = ([SD baseline^2^ + SD final^2^] − [2 × *r* × SD baseline × SD final]). The correlation coefficient (*r*) values were determined according to the Cochrane Handbook (Cumpston et al. 2019). For studies lacking reported change data, mean and SD values were calculated according to the Cochrane Handbook guidelines (Cumpston et al. 2019), and standard errors were converted to SDs (Higgins et al. 2011). Results reported in various measurement units were standardized. Values for TG, LDL‐C, HDL‐C, and non‐HDL‐C are commonly reported in mg/dL or mmol/L. For the conversion of these values from mmol/L to mg/dL, a factor of 38.6 was applied to LDL‐C, HDL‐C, and TC values, whereas TG values required a multiplication by 88.6 (*Circulation* 2023).

We used a random‐effects model, which takes into account variances between studies when determining the overall impact size (Higgins et al. 2003). Additionally, we used the *I* ^*2*^ statistic and Cochran's Q test to examine between‐study heterogeneity (Higgins et al. 2003). High between‐study heterogeneity was defined as *I* ^2^ > 40% or *p* < 0.05. We conducted a sensitivity analysis to test the robustness of the findings (Patsopoulos et al. 2008). To detect publication bias, we utilized funnel plots, Begg's, and Egger's tests (Egger et al. 1997). The traditional two‐stage approach requires calculating individual dose–response curves for each study before combining the results, which may introduce “two‐stage error” (Crippa and Orsini 2016). The robust error meta‐regression (REMR) model integrates all data within a single‐stage framework, reducing parameter assumptions and enhancing estimation accuracy. Additionally, the REMR model can account for within‐study correlations using robust standard errors, thereby mitigating issues that traditional methods may overlook. Therefore, we employed the REMR model for dose–response analysis (Xu and Doi 2018), estimating the impact of Omega‐3 fatty acids intake on lipid profiles and plaque volume at the 10th, 50th, and 90th percentiles (Crippa and Orsini 2016; Orsini et al. 2012). All analyses were conducted using STATA 15.1 software, with statistical significance set at *p* < 0.05.

### 2.6 Grading of Recommendation, Assessment, Development, and Evaluation (GRADE) Assessment of Evidence Quality

The outcome measures were assessed for evidence quality using the GRADE system (Guyatt et al. 2008) that included five downgrade and three upgrade factors. Since the studies included in this review were all RCTs, representing the highest evidence level, no further upgrades were necessary. The evidence quality was graded into four levels: high, moderate, low, and very low to reflect factors such as indirectness, imprecision, bias, inconsistency, and potential publication bias.

## 3 Results

### 3.1 Literature Search

**FIGURE 1:**
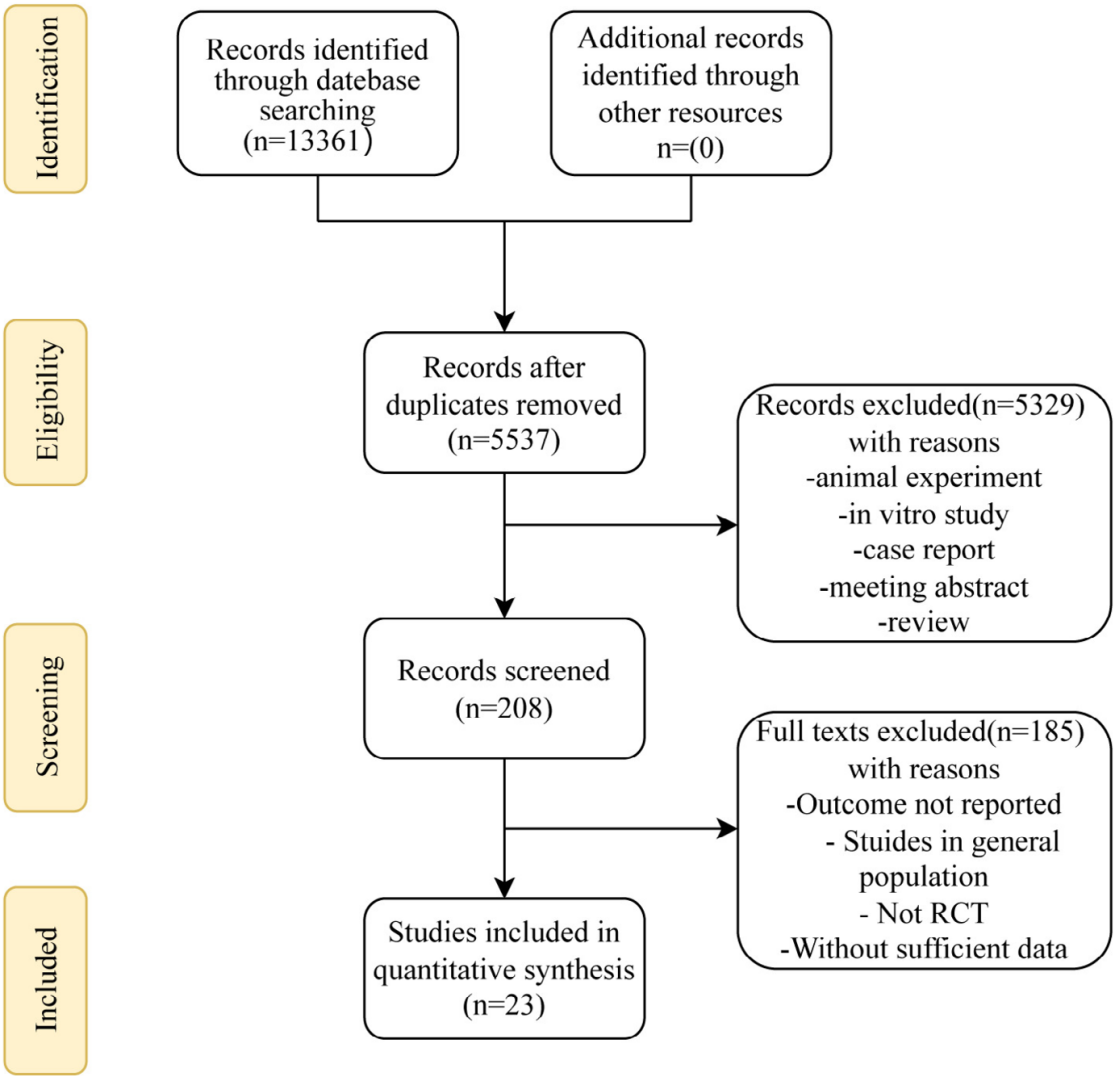
Flowchart of the study selection process for randomized controlled trials (RCT) inclusion in the systematic review of the effects of omega‐3 fatty acids intake on lipid metabolism and plaque volume in patients with coronary heart disease.

The flowchart in Figure 1 illustrates the methodology utilized for analysis in this study. Only studies that adhered to the established criteria for inclusion were considered for this review. Initially, the literature search yielded 13,361 articles that were deemed potentially relevant. In the initial phase of the review process, which involved examining the titles and abstracts, 7824 articles were removed from consideration due to duplication. Subsequently, an additional 5329 articles were ruled out as they did not meet the criteria of being human randomized controlled trials or were identified as conference abstracts, review articles, or as content not pertinent to the subject matter of this review. We further reviewed 208 articles, and finally, 23 studies (Sacks et al. 1995; von Schacky et al. 1999; Johansen et al. 1999; Seierstad et al. 2005; Lee et al. 2006; Ishikawa et al. 2010; Nishio et al. 2014; Ahn et al. 2016; Niki et al. 2016; Tani et al. 2017, 2019, 2020; Watanabe et al. 2017; Alfaddagh et al. 2017; Samavat et al. 2018; Saleh‐Ghadimi et al. 2019; Sugizaki et al. 2020; Budoff et al. 2020; Ćurić et al. 2021; Jafari Salim et al. 2017; Sawada et al. 2016; Nakao et al. 2024; Kita et al. 2020) were included in our meta‐analysis. The inter‐rater agreement on the included studies was robust (Cohen's kappa = 0.86) as shown in Table S3. A list of partial full‐text studies that were excluded is shown in Table S4 (Mostowik et al. 2013; Birudaraju et al. 2020; Cawood et al. 2010; Yuan et al. 2021; Mahdavi‐Roshan et al. 2021; Filion et al. 2010; Lavie et al. 2009; Сусеков 2021; von Schacky et al. 2001; Singh et al. 2012).

### 3.2 Study Characteristics

**FIGURE 2:**
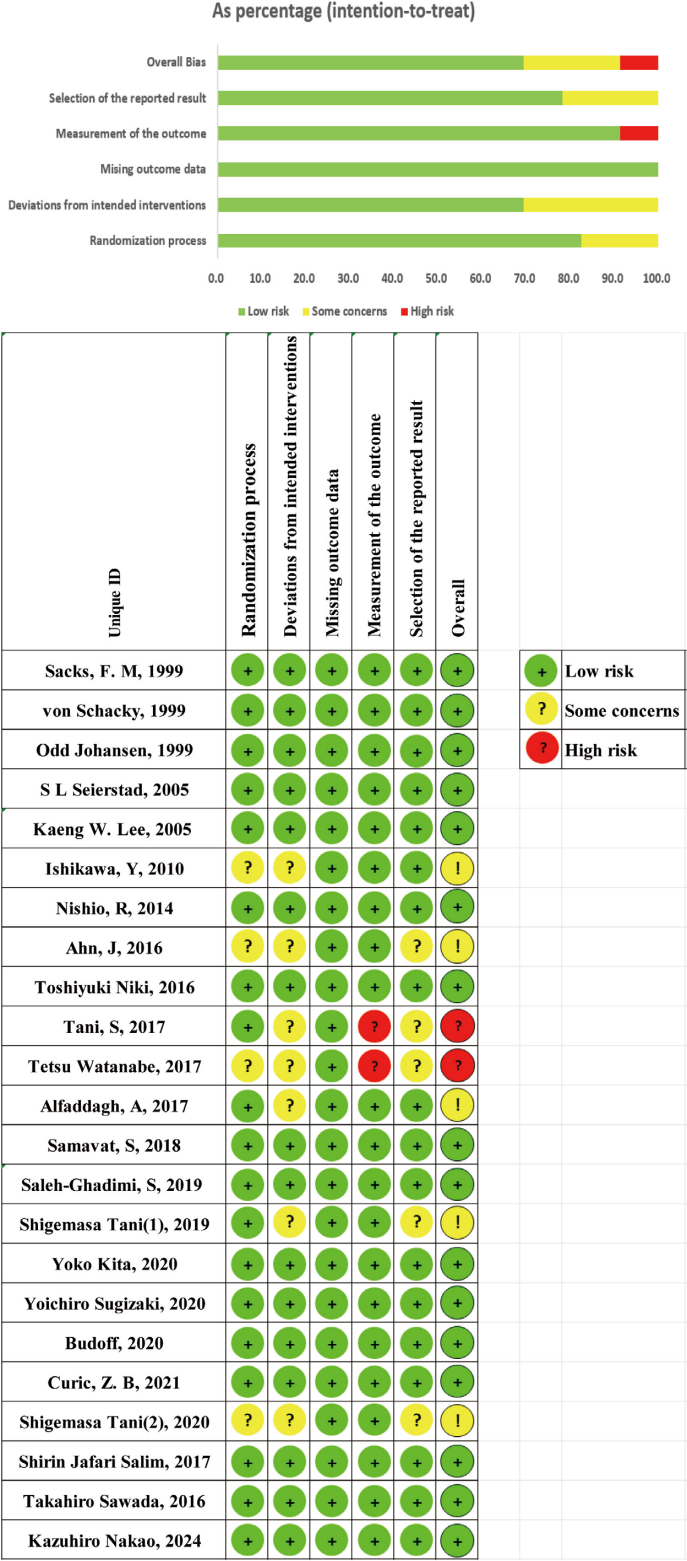
Performance of each type of bias in all studies.

The analysis included 23 studies with 2061 participants. Details of the study characteristics are outlined in Table 1 (Sacks et al. 1995; von Schacky et al. 1999; Johansen et al. 1999; Seierstad et al. 2005; Lee et al. 2006; Ishikawa et al. 2010; Nishio et al. 2014; Ahn et al. 2016; Niki et al. 2016; Tani et al. 2017, 2019, 2020; Watanabe et al. 2017; Alfaddagh et al. 2017; Samavat et al. 2018; Saleh‐Ghadimi et al. 2019; Sugizaki et al. 2020; Budoff et al. 2020; Ćurić et al. 2021; Jafari Salim et al. 2017; Sawada et al. 2016; Nakao et al. 2024; Kita et al. 2020), with all studies being parallel randomized controlled trials. The included studies enrolled participants numbering between 15 and 122, with the mean patient age ranging from 54.8 ± 7.8 to 75.3 ± 8.81 years. Intervention durations ranged from 6 weeks to 5 years. Of the studies, 18 reported on HDL‐C, 18 on LDL‐C, 20 on TG, 19 on TC, and six on changes in coronary atherosclerotic plaque volume. Sixteen studies (69.6%) were rated as having a low risk of bias across all domains, while five studies (21.7%) raised some concerns in certain domains, and two studies (8.7%) were judged to be at high risk of bias. The most frequently observed sources of bias were deviations from intended interventions, issues in the randomization process, and selective reporting of results (Figure 2).

**TABLE 1:** Characteristics of the randomized controlled studies.

Authors	Country	Year	Population	Age (mean + SD)	Total/male/female	Intervention	Follow up	Outcome
Sacks et al. (1995)	USA	1995	coronary artery disease	T: 62 (7)	T: 31/29/2	6 g of n‐3 fatty acids	28 months	TG, TC, HDL‐C, LDL‐C
				C: 62 (7)	C: 28/26/2			
von Schacky et al. (1999)	Germany	1999	coronary artery disease	T: 58.7 (9.7)	T: 111/91/20	6 g EPA + DHA	21 months	TG, TC, HDL‐C, LDL‐C
				C: 58.9 (8.1)	C: 112/88/32			
Johansen et al. (1999)	USA	1999	coronary artery disease	T: 57.3 (22.96)	T: 23/18/3	0.45gEPA + 0.39gDHA	6 months	TG, TC, HDL‐C
				C: 57.7 (24.44)	C: 31/21/10			
Seierstad et al. (2005)	Norwegian	2005	coronary artery disease	T: 58 (20.74)	T: 20/17/3	fish oil	1.5 months	TG, TC, HDL‐C, LDL‐C
				C: 63 (17.037)	C: 19/18/1			
Lee et al. (2006)	UK	2005	acute coronary syndrome	T: 59 (10)	T: 37/35/2	Ω3 PUFAs supplementation	3 months	TG, TC, HDL‐C, LDL‐C
				C: 55 (10)	C: 40/36/4			
Ishikawa et al. (2010)	Japan	2010	coronary artery disease	T: 60.8 (8.5)	T: 96/54/42	EPA	5 years	TG, HDL‐C, LDL‐C
				C: 60.5 (8.5)	C: 77/48/29			
Nishio et al. (2014)	Japan	2014	coronary artery disease	T: 61 (12.6)	T: 15/13/2	1.8gEPA	9 months	TG, TC, HDL‐C, LDL‐C
				C: 63.8 (9.5)	C: 15/13/2			
Ahn et al. (2016)	Korea	2016	coronary artery disease	T: 59.6 (9.1)	T: 38/24/14	1.395gEPA + 1.125gDHA	12 months	TG, TC, HDL‐C, LDL‐C, plaque
				C: 60.7 (0.8)	C: 36/26/10			
Niki et al. (2016)	Japan	2016	coronary artery disease	T: 68.1 (10.1)	T: 48/21/27	EPA	6 months	Plaque
				C: 69.4 (10.7)	C: 47/19/28			
Tani et al. (2017)	Japan	2017	coronary artery disease	T: 68 (11)	T: 53/49/4	1.8 g EPA	6 months	TG, TC, HDL‐C, LDL‐C
				C: 66 (11)	C: 53/44/9			
Watanabe et al. (2017)	Japan	2017	coronary artery disease	T: 67 (10)	T: 97/67/30	1.8gEPA	8 months	TG, TC, HDL‐C, LDL‐C, plaque
				C: 68 (10)	C: 96/45/51			
Alfaddagh et al. (2017)	USA	2017	coronary artery disease	T: 62.4 (7.8)	T: 122/104/18	1.86gEPA + 1.5gDHA	30 months	TG, TC, HDL‐C, LDL‐C, plaque
				C: 62.6 (7.5)	C: 97/84/13			
Saleh‐Ghadimi et al. (2019)	Iran	2019	coronary artery disease	T: 55.67 (6.9)	T: 21/19/2	2.5 g ALA	2.5 months	TG, TC, HDL‐C, LDL‐C
				C: 54.80 (7.8)	C: 19/17/2			
Samavat et al. (2018)	Iran	2018	coronary artery disease	T: 56.19 (1.35)	T: 21/NA/NA	0.72gEPA + 0.48gDHA	2 months	TG, TC
				C: 57.86 (1.45)	C: 21/NA/NA			
Tani et al. (2019)	Japan	2019	coronary artery disease	T: 67.5 (10.1)	T: 50/46/4	1.8gEPA	6 months	TG, TC, HDL‐C, LDL‐C
				C: 67.3 (10.4)	C: 50/42/8			
Kita et al. (2020)	Japan	2020	acute coronary syndrome	T1: 64.2 (10.1)	T1: 31/24/7	1.8gEPA	8 months	TG, TC, HDL‐C, LDL‐C
				T2: 67 (8.15)	T2: 35/28/7	0.93gEPA + 0.75gDHA		
				C: 63 (13.33)	C: 31/27/4			
Sugizaki et al. (2020)	Japan	2020	coronary artery disease	T: 70.8 (7.7)	T: 21/17/4	1.8gEPA + 10 mg rosuvastatin	12 months	TG, TC, HDL‐C, LDL‐C
				C: 75.3 (8.81)	C: 21/16/5			
Budoff et al. (2020)	Turkey	2020	coronary artery disease	T: 56.5 (8.9)	T: 31/17/14	IPE	18 months	Plaque
				C: 58.3 (8.6)	C: 37/20/17			
Ćurić et al. (2021)	Croatia	2021	coronary artery disease	T: NA (NA)	T: 20/NA/NA	n‐3 PUFA‐enriched hen eggs	3 months	TG, TC, HDL‐C, LDL‐C
				C: NA (NA)	C: 20/NA/NA			
Tani et al. (2020)	Japan	2020	coronary artery disease	T: 63.9 (10.6)	T: 30/28/2	1.8gEPA	6 months	TG, TC, HDL‐C, LDL‐C
				C: 65.5 (10.9)	C: 30/25/5			
Jafari Salim et al. (2017)	Iran	2017	coronary artery disease	T: 54.86 (6.05)	T: 21/21/0	0.72gEPA + 0.48DHA	2 months	TG, TC, HDL‐C, LDL‐C
				C: 57.76 (6.26)	C: 21/21/0			
Sawada et al. (2016)	Japan	2016	coronary artery disease	T: 67.8 (9.1)	T: 54/44/10	1.8gEPA	6 months	TG, TC, HDL‐C, LDL‐C
				C: 68.9 (8.8)	C: 53/43/10			
Nakao et al. (2024)	Japan	2024	coronary artery disease	T1: 67.6 (9.1)	T1: 28/24/4	0.93gEPA + 0.75DHA	12 months	TG, TC, HDL‐C, LDL‐C, Plaque
				T2: 69 (8.1)	T2: 26/22/4	1.86gEPA + 1.5gDHA	12 months	TG, TC, HDL‐C, LDL‐C, Plaque
				C: 63.8 (10.5)	C: 27/33/4			

Abbreviations: C, control group; DHA, docosahexaenoic acid; EPA, eicosapentaenoic acid; HDL‐C, high‐density lipoprotein; IPE, Icosapent ethyl; LDL‐C, low‐density lipoprotein; T, treatment group; TC, total cholesterol; TG, triglycerides.

### 3.3 Relationship Between Omega‐3 Fatty Acids Intake and TG Levels

**FIGURE 3:**
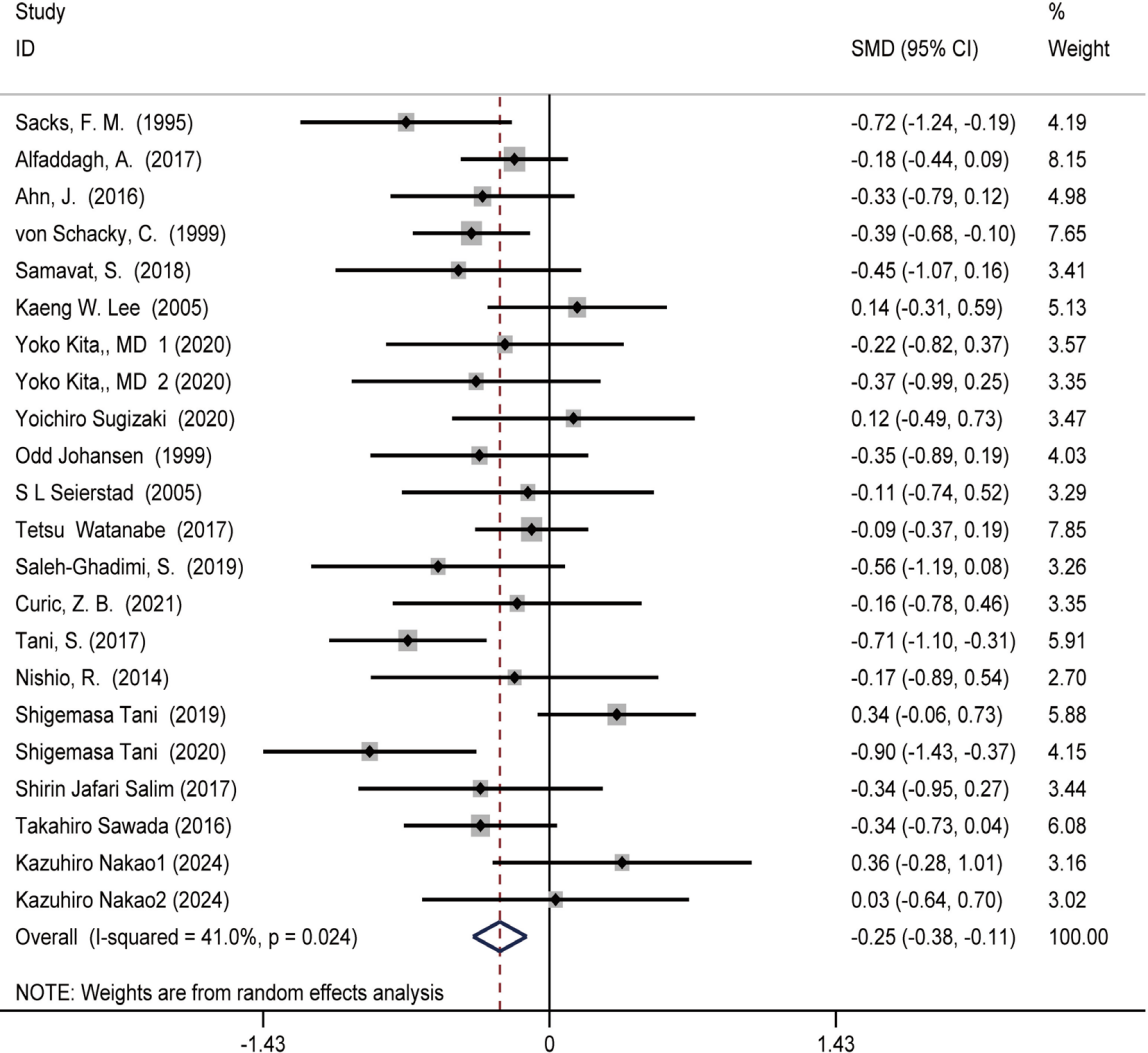
Forest plot illustrating the standardized mean difference and 95% confidence interval for the effects of omega‐3 fatty acids intake on triglyceride (TG) levels (SMD = −0.25, 95% CI = [−0.38, −0.11], *I* ^2^ = 41%, *p* = 0.024). CI, confidence interval; SMD, standardized mean difference.

The studies on omega‐3 fatty acids intake and TG levels comprised 20 reports involving 24 treatment groups and 1685 participants (intervention = 888, control = 797). Moderate‐quality evidence showed a statistically significant reduction in TG levels with omega‐3 fatty acids supplementation compared with the control group (SMD = −0.25 (−0.38, −0.11), *p* = 0.024, *I* ^2^ = 41%) (Figure 3). Subgroup analysis showed the following short‐term intervention results: SMD = −0.3 (−0.54, 0.06), *p* = 0.007, *I* ^2^ = 58.5%. Long‐term intervention results were as follows: SMD = −0.25 (−0.35, 0.07), *p* = 0.363, *I* ^2^ = 8.5%. The ≤ 2 g/day intervention results were as follows: SMD = −0.22 (−0.39, 0.06), *p* = 0.016, *I* ^2^ = 47.4%. The > 2 g/day intervention results were as follows: SMD = −0.31 (−0.54, 0.09), *p* = 0.0306, *I* ^2^ = 47.4%. The results of the remaining subgroup analyses are presented in Table S5.

### 3.4 Relationship Between Omega‐3 Fatty Acids Intake and HDL‐C Levels

**FIGURE 4:**
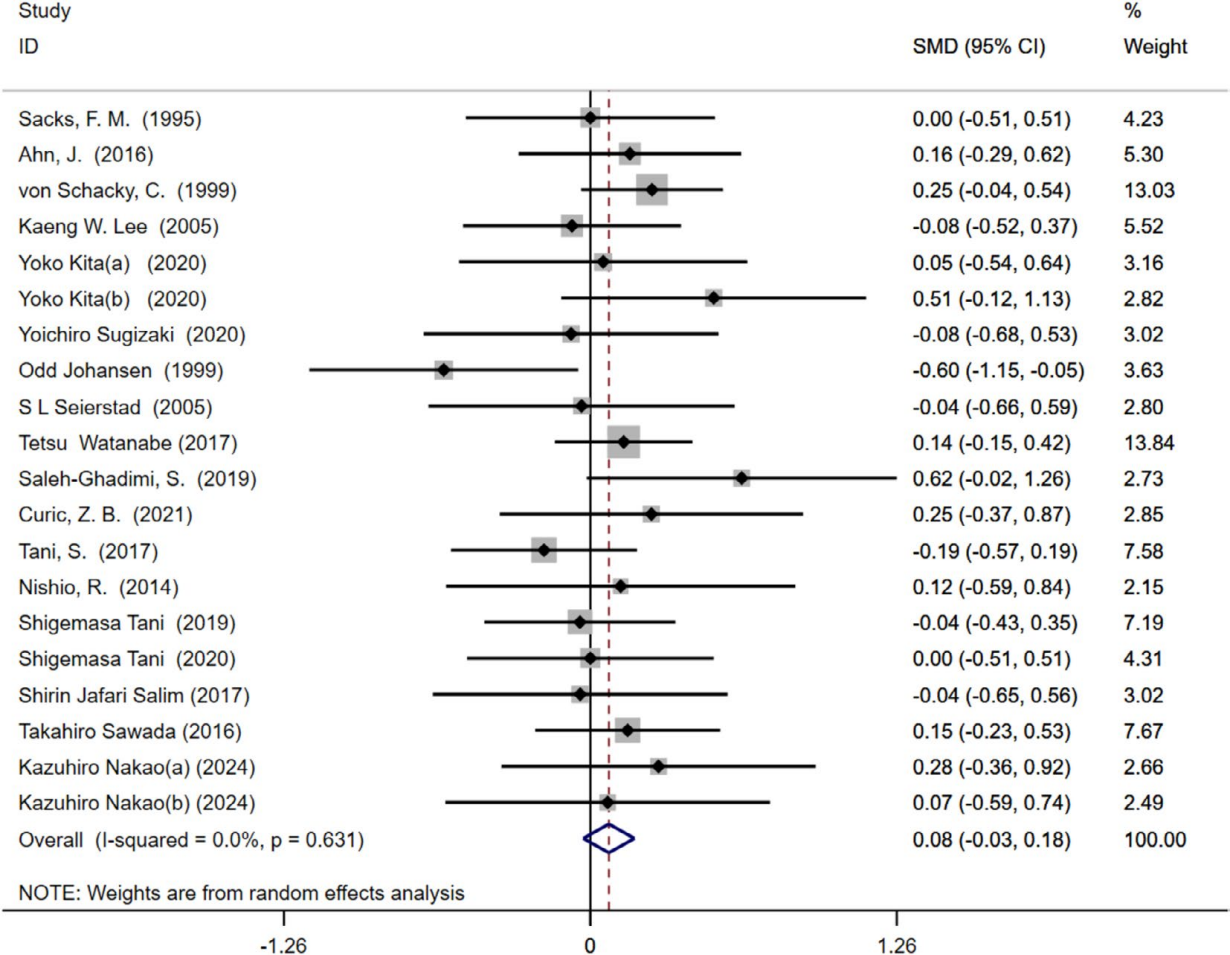
Forest plot illustrating the standardized mean difference and 95% confidence interval for the effects of omega‐3 fatty acids intake on high‐density lipoprotein cholesterol (HDL‐C) levels (SMD = 0.08, 95% CI = [−0.03, 0.18], *I* ^2^ = 0%, *p* = 0.631). CI, confidence interval; SMD, standardized mean difference.

Our analysis of omega‐3 fatty acids intake on HDL‐C levels included 18 studies featuring 20 treatment arms and a total of 1424 participants (intervention group: 745, control group: 679). Moderate‐quality evidence showed no significant effect of omega‐3 fatty acids supplementation on HDL‐C levels relative to the control group (SMD = 0.08 (−0.03, 0.18), *I* ^2^ = 0%, *p* = 0.631) (Figure 4). Subgroup analysis showed that long‐term intervention (> 6 monthes) could increase HDL‐C levels, with a combined effect size as follows: SMD = 0.16 (0.02, 0.31), *I* ^2^ = 0%, *p* = 0.968. Conversely, short‐term intervention (≤ 6 monthes) and dosage subgroups showed no significant effects (Table S5).

### 3.5 Relationship Between Omega‐3 Fatty Acids Intake and LDL‐C Levels

**FIGURE 5:**
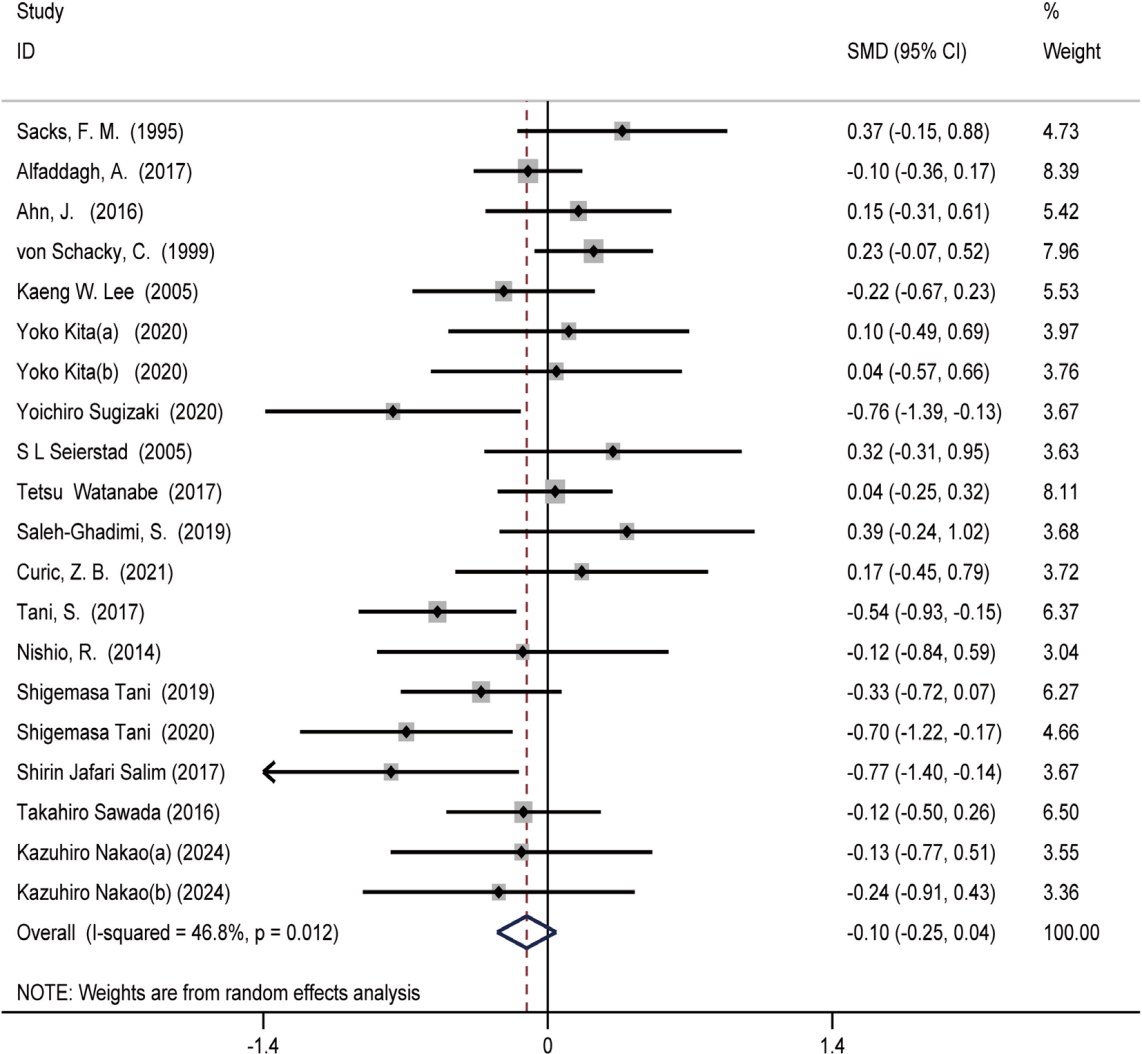
Forest plot illustrating the standardized mean difference and 95% confidence interval for the effects of omega‐3 fatty acids intake on low‐density lipoprotein cholesterol (LDL‐C) levels (SMD = −0.1, 95% CI = [−0.25, 0.04], *I* ^2^ = 46.8%, *p* = 0.012). CI, confidence interval; SMD, standardized mean difference.

The studies on omega‐3 fatty acids intake and LDL‐C levels included 18 reports involving 20 treatment groups, comprising 1589 participants (intervention = 844, control = 745). Moderate‐quality evidence showed no significant influence of omega‐3 fatty acids intake on LDL‐C levels (SMD = −0.1 (−0.25, 0.04), *I* ^2^ = 46.8%, *p* = 0.012) (Figure 5). However, in the subgroup analysis, we found that the pure EPA group (SMD = −0.29 (−0.51, 0.07), *I* ^2^ = 46.4%, *p* = 0.071) was better than the EPA + DHA (SMD = −0.02 (−0.21, 0.17), *I* ^2^ = 36.5%, *p* = 0.126) mixed group. Variations in disease states, intervention durations, and doses may account for the heterogeneity observed among the included studies (Table S5).

### 3.6 Relationship Between Omega‐3 Fatty Acids Intake and TC Levels

The studies on omega‐3 fatty acids intake and TC levels included 19 reports involving 21 treatment groups, comprising 1466 participants (intervention = 766, control = 700).

**FIGURE 6:**
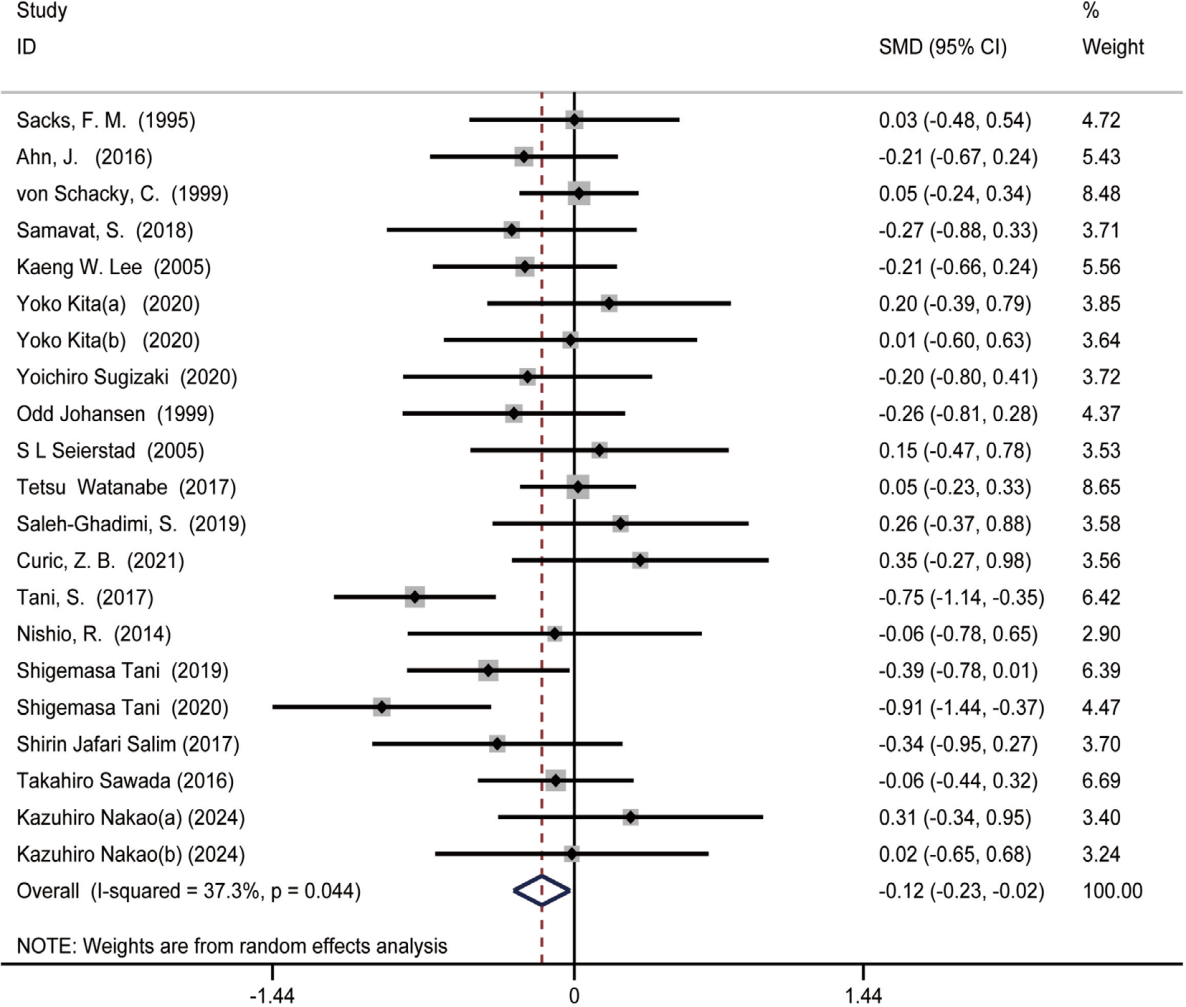
Forest plot illustrating the standardized mean difference and 95% confidence interval for the effects of omega‐3 fatty acids intake on total cholesterol (TC) levels (SMD = −0.12, 95% CI = [−0.23, −0.02], *I* ^2^ = 37.3%, *p* = 0.044). SMD, CI, confidence interval; standardized mean difference.

Moderate‐quality evidence showed a statistically significant reduction in TC levels with omega‐3 fatty acids supplementation compared to the control group (SMD = −0.12 (−0.23, −0.02), *I* ^2^ = 37.3%, *p* = 0.044) (Figure 6). Subgroup analysis showed that short‐term intervention (SMD = −0.25 (−0.47, −0.03), *I* ^2^ = 52.4%, *p* = 0.021) and pure EPA group (SMD = −0.29 (−0.55, −0.03), *I* ^2^ = 62.5%, *p* = 0.009) could reduce TC levels (Table S5).

### 3.7 Relationship Between Omega‐3 Fatty Acids Intake and Coronary Artery Plaque Volume

**FIGURE 7:**
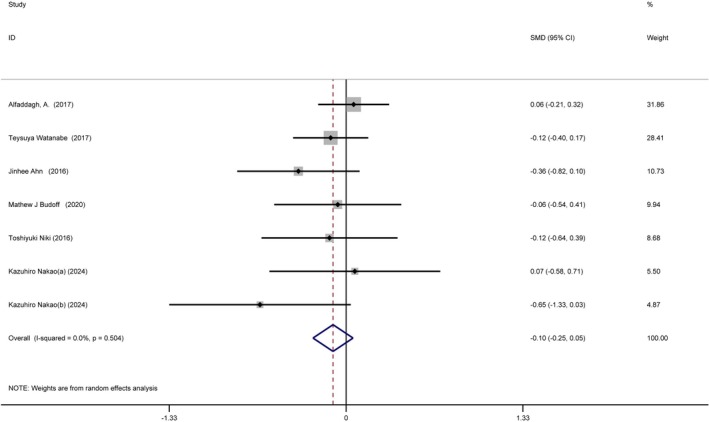
Forest plot illustrating the standardized mean difference and 95% confidence interval for the effects of omega‐3 fatty acids intake on coronary artery plaque volume (SMD = −0.10, 95% CI = [−0.25, 0.05], *I* ^2^ = 0%, *p* = 0.504). CI, confidence interval; SMD, standardized mean difference.

Our analysis included six studies with seven treatment arms and 694 participants, exploring the effect of omega‐3 fatty acids intake on plaque volume changes (intervention = 371, control = 323). Low‐quality evidence revealed no significant effect of omega‐3 fatty acids intake on plaque volume (SMD = −0.10 [−0.25, 0.05], *I* ^2^ = 0%, *p* = 0.504) (Figure 7).

### 3.8 Sensitivity Analysis and Publication Bias

We found that the exclusion of individual studies had no significant impact on the pooled effect size (Figure S1). We assessed the publication bias for the TG, HDL‐C, LDL‐C, and TC outcomes. The funnel plot appeared symmetrical (Figure S2), and both the Egger's test as well as Begg's test had *p*‐values greater than 0.05 (Table S6), indicating no significant publication bias. Given the small sample size of studies addressing coronary atherosclerotic plaques, a funnel plot was not generated.

### 3.9 GRADE Assessment

The GRADE system was used to assess the strength of evidence. For TG, moderate heterogeneity between studies (*I* ^2^ = 41%), possibly due to differences in intervention duration and dosage (e.g., ≤ 6 monthes vs. > 6 monthes, ≤ 2 g/day vs. > 2 g/day), led to downgrading the evidence quality to “moderate”. For HDL‐C, a high loss to follow‐up among study participants, which could introduce bias in the overall effect estimate, led to downgrading the quality of evidence to “moderate”. TC (*p*_nonlinear = 0.004) and LDL‐C (*p*_nonlinear = 0.005) exhibited “J‐shaped” dose–response curves, suggesting attenuation of effects at higher doses and resulting in uncertainty about outcome direction; consequently, the evidence was downgraded to “moderate.” For coronary plaque volume, serious indirectness was identified, attributed to measurement biases (CCTA/IVUS), the inclusion of only six studies, methodological heterogeneity, and a limited range of study populations, thereby restricting applicability to broader clinical practice. The quality of evidence for plaque volume was rated as “low” (Table S7).

### 3.10 Nonlinear Dose–Response Analysis Employing the REMR Model

**FIGURE 8:**
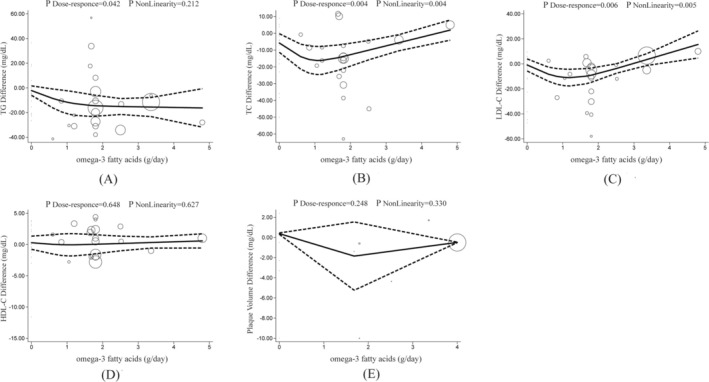
Nonlinear dose–response analysis of the effects of omega‐3 fatty acids intake on lipid metabolism and arterial plaque volume in patients with coronary heart disease (REMR model analysis). Individual randomized clinical trial (RCT) are represented by circles, with the size of each circle corresponding to its weight in the overall analysis. The solid lines represent the estimated dose–response relationships for omega‐3 fatty acids intake on the circulating levels of (A) triglyceride (TG), (B) total cholesterol (TC), (C) low‐density lipoprotein cholesterol (LDL‐C), (D) high‐density lipoprotein cholesterol (HDL‐C), and (E) arterial plaque volume. The dashed lines represent the 95% confidence intervals (CI). REMR, robust error meta‐regression.

Employing the one‐stage REMR model, we identified a linear dose–response relationship between the intervention dose of omega‐3 fatty acids and the mean change in TG levels (*p*_linear = 0.042) (Figure 8A). The changes in mean values for TC (Figure 8B, *p*_nonlinear = 0.004) and LDL‐C (Figure 8C, *p*_nonlinear = 0.005) exhibited a “J” shape relationship. Specifically, omega‐3 fatty acids supplementation at doses of 1–2 g/day may be more effective in reducing circulating levels of TC and LDL‐C. There was no nonlinear relationship between the mean value changes in HDL‐C (Figure 8D, *p*_nonlinear = 0.627) and plaque volume (Figure 8E, *p*_nonlinear = 0.330).

## 4 Discussion

In this study, we performed a one‐stage dose–response meta‐analysis to examine the effects of omega‐3 fatty acids intake on lipid profiles and plaque characteristics in patients with. The GRADE assessment indicated that the quality of evidence for improvements in TGs and total cholesterol (TC) was moderate, supporting the use of omega‐3 fatty acids as adjunctive therapy for lipid management in patients with CHD. However, omega‐3 fatty acids did not significantly affect HDL‐C or LDL‐C levels. Additionally, the low‐quality evidence regarding plaque volume suggests that current data are insufficient to recommend omega‐3 fatty acids for promoting plaque regression. A further nonlinear dose–response meta‐analysis revealed a “J”‐shaped relationship between omega‐3 fatty acids consumption and TC and LDL‐C levels, suggesting a need for determining optimal intake strategies.

The pooled analysis showed that omega‐3 fatty acids supplementation significantly reduced TG levels in patients with CHD, with a dose‐dependent effect. This finding is consistent with previous studies by Balk et al. (2006) and Wang et al. (2023), further confirming the role of TG as an independent risk factor for CHD (Faergeman et al. 2009). Previous studies have suggested that a daily intake of 3–4 g EPA + DHA can significantly reduce TG levels by 20%–50% in individuals with serum TG levels exceeding 150 mg/dL (Backes et al. 2016; Kastelein et al. 2014). This dose‐dependent effect provides important dosing guidance for clinical treatment. Omega‐3 fatty acids demonstrate a lower incidence of side effects and drug interactions compared to fibrates and niacin, while still effectively reducing TG levels (M et al. 2011). The 2019 American Diabetes Association guidelines emphasize this point, stating that the combination of statins with fibrates or niacin is not recommended for preventing cardiovascular events (*Diabetes Care* 2018). Clinical studies have indicated that niacin and fibrates do not show significant advantages in reducing cardiovascular event rates in patients with CHD and often cause discomfort such as gastrointestinal issues, liver dysfunction, and hyperuricemia (Group THTC 2014; Regemorter et al. 2022). The advantages of omega‐3 fatty acids supplementation position them as a compelling choice for treating dyslipidaemia in patients with CHD, with the added benefit of fewer potential side effects and medication interactions (Bhatt et al. 2019; Ito 2015). Although the exact mechanism by which omega‐3 fatty acids intake reduces serum TG levels remains elusive, it is believed to include effects on lipid synthesis and metabolism, as well as promoting fatty acid oxidation (Le Jossic‐Corcos et al. 2005). Omega‐3 fatty acids are speculated to reduce TG levels by inhibiting key liver enzymes in TG synthesis and upregulating lipoprotein lipase gene expression, thereby accelerating the clearance of TG from very low‐density lipoprotein (LDL) and chylomicrons (Bays et al. 2008). Our present review offers a new perspective on using omega‐3 fatty acids for treating CHD. Nonetheless, further investigation is required to clarify the specific mechanisms of TG level reduction, explore dose–response relationships, and evaluate long‐term effectiveness in various patient groups.

Our study is the first to reveal a “J‐shaped” curve relationship between omega‐3 fatty acids supplementation and TC and LDL‐C levels. Specifically, omega‐3 intake below 2 g/day significantly decreased TC (SMD = −0.15) and LDL‐C (SMD = −0.17), potentially due to EPA suppressing hepatic very low‐density lipoprotein (VLDL) synthesis and promoting fatty acid β‐oxidation (Bays et al. 2008). This intake range is consistent with the majority of clinical guidelines, including the American Heart Association (AHA) recommendation of 1–2 g/day of EPA + DHA for secondary prevention of cardiovascular diseases (Siscovick et al. 2017). When the intake exceeds 2 g/day, the reductions in TC and LDL‐C attenuate or may even reverse. Potential mechanisms include high doses of omega‐3 fatty acids inhibiting hepatic LDL receptor expression and reducing LDL‐C clearance (Harris 1997), as well as excessive polyunsaturated fatty acid intake promoting lipid peroxidation and the formation of small, dense LDL particles (Bays et al. 2008). Consequently, in patients with coronary heart disease, an initial omega‐3 intake of less than 2 g/day is recommended, with routine monitoring of LDL‐C levels; if additional TG lowering is required (e.g., in cases of severe hypertriglyceridemia), dose escalation should be approached cautiously while considering the potential risk of LDL‐C increase.

We found that omega‐3 fatty acid intake had no significant effect on HDL‐C levels or plaque volume. Studies have shown that oxidized LDL‐C causes endothelial cell damage, leading to plaque formation, and plaque rupture is a primary causative risk for acute coronary events (Stefanadis et al. 2017). In patients with CHD, plaque volume and its stability are key determinants of the risk for adverse cardiovascular events (Mortensen et al. 2020). Kashiyama et al. (2011) have demonstrated that reduced EPA levels are accompanied by an increased susceptibility for arterial plaque formation. Additionally, different components of omega‐3 fatty acid supplements may have significant differences in outcomes. Urabe et al. (2013) discovered a link between serum EPA levels and coronary artery plaques, while no significant association was found for DHA levels. Prior meta‐analyses have indicated that omega‐3 fatty acids supplementation may lead to a reduction in arterial plaque volume (SMD = −0.18; 95% CI [−0.31, −0.05]) (Gao et al. 2022). However, the meta‐analysis by Gao et al. had issues such as including nonrandomized controlled trials (Amano et al. 2011) and errors in data extraction (Alfaddagh et al. 2019). Therefore, we excluded the relevant included literature. Considering the limited effect of omega‐3 fatty acids intake on arterial plaques, further high‐quality studies may be needed to confirm its mechanism of action and clinical efficacy.

Although this study primarily focused on surrogate endpoints such as lipid profiles and coronary plaque volume, there remains some controversy over whether TG reduction can translate into improvements in clinical hard endpoints, such as myocardial infarction, stroke, or mortality. Evidence from multiple large randomized controlled trials suggests that, under specific populations and intervention protocols, TG reduction may correlate with a lower risk of cardiovascular events. For example, the REDUCE‐IT trial demonstrated that in populations at high cardiovascular risk, high‐purity EPA at a dose of 4 g/day reduced TG levels by approximately 19% and significantly lowered the risk of major adverse cardiovascular events, myocardial infarction, stroke, and cardiovascular death (Bhatt et al. 2019). Similarly, the JELIS trial showed that EPA combined with statin therapy reduced TG levels and coronary events in Japanese patients with hypercholesterolemia, with particularly greater benefits observed in subgroups with higher baseline TG levels (Yokoyama et al. 2007). However, the STRENGTH (Nicholls et al. 2020) and OMEMI (Kalstad et al. 2021) trials failed to demonstrate cardiovascular benefits associated with TG reduction, possibly due to variations in omega‐3 formulation (EPA + DHA vs. pure EPA), purity, dose, and population heterogeneity. In certain trials, DHA may have contributed to an increase in LDL‐C, potentially offsetting the benefits of TG reduction. These findings suggest that although TG reduction may be a necessary condition for cardiovascular benefit, it is not sufficient, and the actual efficacy may also depend on mechanisms such as the anti‐inflammatory, antioxidant, and plaque‐stabilizing effects of omega‐3 fatty acids (Budoff et al. 2020). Future high‐quality randomized controlled trials are needed to further elucidate the effects of different types of omega‐3 fatty acids on clinical outcomes across diverse populations, thereby informing personalized treatment strategies.

Reducing TG levels, an important goal in the secondary prevention of cardiovascular disease, may lead to better clinical outcomes in specific groups, especially in individuals with hypertriglyceridemia or heightened inflammatory risk. Research suggests that when conventional lipid‐lowering therapies become ineffective for individuals with elevated TG levels, combining omega‐3 fatty acids intake with conventional treatment may yield unexpected benefits (Saito et al. 2008). Moreover, the European Atherosclerosis Society and the European Society of Cardiology have endorsed the use of omega‐3 fatty acids intake in combination with statins for high‐risk patients with TG levels ranging from 135 to 499 mg/dL, graded as a IIa recommendation. Yuan et al. (2021) found that omega‐3 fatty acids intake may benefit patients with postacute myocardial infarction by affecting lipid metabolism and endothelial function. The 2019 American Heart Association Annual Scientific Conference highlighted that pure EPA is cost‐effective and may reduce long‐term medical expenses. This prompts a reevaluation of the substantial role omega‐3 fatty acids may play in atherosclerosis prevention and in managing TG and cholesterol levels, potentially providing clinicians with novel strategies for CHD prevention and management.

Our study has several limitations. First, our analysis was based on aggregate data, which precluded adjustment for individual‐level confounders and may have contributed to heterogeneity in effect estimates. Second, residual confounding factors—such as smoking status, physical activity, and dietary habits—may have influenced lipid levels and plaque progression. Additionally, variations in measurement methods and outcome definitions across studies could have further contributed to heterogeneity. Moreover, the included studies primarily enrolled middle‐aged and elderly patients, limiting the generalizability of our findings to younger CHD populations. Finally, due to insufficient data, we were unable to perform subgroup analyses based on sex. Future research should prioritize individual participant data meta‐analyses to overcome these limitations and explore personalized omega‐3 dosing strategies across diverse populations.

## 5 Conclusion

In patients with CHD, dietary supplementation with omega‐3 fatty acids has a beneficial effect on lowering circulating TG and TC levels, particularly within the first 6 months of treatment. Additionally, our dose–response analysis revealed a “J”‐shaped correlation between omega‐3 fatty acids intake and TC as well as LDL‐C serum levels. Our findings underscore the importance of tailoring omega‐3 fatty acids dosage to individual patient needs in clinical settings.

## Author Contributions

**Chunyu Zhang:** conceptualization (equal). **Yulei Xie:** conceptualization (equal). **Jie Zhou:** methodology (equal). **Chun Zhang:** data curation (equal). **Qilang Xiang:** formal analysis (equal). **Yi Zhong:** methodology (equal). **Juan Xiao:** methodology (equal). **Jian Feng:** writing – review and editing (equal). **Bin Liao:** supervision (equal). **Xuxin Chen:** methodology (equal). **Li Deng:** writing – review and editing (equal).

## Ethics Statement

This review was conducted according to the PRISMA (Preferred Reporting Items for Systematic Reviews and Meta‐Analysis) guidelines.

## Consent

The authors have nothing to report.

## Conflicts of Interest

The authors declare no conflicts of interest.

## Supporting information


**Figure S1.** Sensitivity analysis results of different outcome indicators.
**Figure S2.** Funnel plots for different outcome indicators.
**Table S1.** PRISMA checklist 2020.
**Table S2.** Search terms employed in the literature search.
**Table S3.** Calculation of the raw proportion of agreement by Cohen’s Kappa statistic.
**Table S4.** Reason for exclusion of retrieved articles.
**Table S5.** Subgroup analysis of outcomes by time, dose, type, country, mean age and disease state.
**Table S6.** Results of Egger’s Regression Test and Begg’s Rank Correlation Test for various outcome measures.
**Table S7.** GRADE evidence table for the effects of omega‐3 fatty acids on blood lipids and plaques in patients with coronary atherosclerotic heart.

## Data Availability

The data that support the findings of this study are available from the corresponding author (J.F.) upon reasonable request.
