# Supplementary material for: Effects of Omega‐3 Fatty Acids Intake on Lipid Metabolism and Plaque Volume in Patients With Coronary Heart Disease: A Systematic Review and Dose–Response Meta‐Analysis of Randomized Clinical Trials
Source: Food Sci Nutr. 2025 Jun 2;13(6):e70372. doi: 10.1002/fsn3.70372 (PMC12129820; doi:10.1002/fsn3.70372)
Supplement: Supplementary file 1 — Figure S1. Sensitivity analysis results of different outcome indicators. Figure S2. Funnel plots for different outcome indicators. Table S1. PRISMA checklist 2020. Table S2. Search terms employed in the literature search. Table S3. Calculation of the raw proportion of agreement by Cohen’s Kappa statistic. Table S4. Reason for exclusion of retrieved articles. Table S5. Subgroup analysis of outcomes by time, dose, type, country, mean age and disease state. Table S6. Results of Egger’s Regression Test and Begg’s Rank Correlation Test for various outcome measures. Table S7. GRADE evidence table for the effects of omega‐3 fatty acids on blood lipids and plaques in patients with coronary atherosclerotic heart. [file FSN3-13-e70372-s001.docx]

**Effects of omega-3 fatty acids intake on lipid metabolism and plaque volume in patients with coronary heart disease: A Systematic Review and Dose–Response Meta-analysis of Randomized Clinical Trials**

Supplementary Tables S1-S7 and Supplementary Figures S1-S2

**Supplementary Table S1.** PRISMA checklist 2020.

| **Section and Topic** | **Item #** | **Checklist item** | **Location where item is reported** |
| --- | --- | --- | --- |
| **TITLE** | | |  |
| Title | 1 | Identify the report as a systematic review. | Line1-3 |
| **ABSTRACT** | | |  |
| Abstract | 2 | See the PRISMA 2020 for Abstracts checklist. | Line43-56 |
| **INTRODUCTION** | | |  |
| Rationale | 3 | Describe the rationale for the review in the context of existing knowledge. | Line60-66 |
| Objectives | 4 | Provide an explicit statement of the objective(s) or question(s) the review addresses. | Line67-87 |
| **METHODS** | | |  |
| Eligibility criteria | 5 | Specify the inclusion and exclusion criteria for the review and how studies were grouped for the syntheses. | Line88-95 |
| Information sources | 6 | Specify all databases, registers, websites, organisations, reference lists and other sources searched or consulted to identify studies. Specify the date when each source was last searched or consulted. | Line103-107 |
| Search strategy | 7 | Present the full search strategies for all databases, registers and websites, including any filters and limits used. | Line107-113 |
| Selection process | 8 | Specify the methods used to decide whether a study met the inclusion criteria of the review, including how many reviewers screened each record and each report retrieved, whether they worked independently, and if applicable, details of automation tools used in the process. | Line116-124 |
| Data collection process | 9 | Specify the methods used to collect data from reports, including how many reviewers collected data from each report, whether they worked independently, any processes for obtaining or confirming data from study investigators, and if applicable, details of automation tools used in the process. | Line140-147 |
| Data items | 10a | List and define all outcomes for which data were sought. Specify whether all results that were compatible with each outcome domain in each study were sought (e.g. for all measures, time points, analyses), and if not, the methods used to decide which results to collect. | Line140-147 |
|  | 10b | List and define all other variables for which data were sought (e.g. participant and intervention characteristics, funding sources). Describe any assumptions made about any missing or unclear information. | Line142-145 |
| Study risk of bias assessment | 11 | Specify the methods used to assess risk of bias in the included studies, including details of the tool(s) used, how many reviewers assessed each study and whether they worked independently, and if applicable, details of automation tools used in the process. | Line131-137 |
| Effect measures | 12 | Specify for each outcome the effect measure(s) (e.g. risk ratio, mean difference) used in the synthesis or presentation of results. | Line150-163 |
| Synthesis methods | 13a | Describe the processes used to decide which studies were eligible for each synthesis (e.g. tabulating the study intervention characteristics and comparing against the planned groups for each synthesis (item #5)). | Line150-163 |
|  | 13b | Describe any methods required to prepare the data for presentation or synthesis, such as handling of missing summary statistics, or data conversions. | Line150-163 |
|  | 13c | Describe any methods used to tabulate or visually display results of individual studies and syntheses. | Line150-163 |
|  | 13d | Describe any methods used to synthesize results and provide a rationale for the choice(s). If meta-analysis was performed, describe the model(s), method(s) to identify the presence and extent of statistical heterogeneity, and software package(s) used. | Line177-178 |
|  | 13e | Describe any methods used to explore possible causes of heterogeneity among study results (e.g. subgroup analysis, meta-regression). | Line164-169 |
|  | 13f | Describe any sensitivity analyses conducted to assess robustness of the synthesized results. | Line164-169 |
| Reporting bias assessment | 14 | Describe any methods used to assess risk of bias due to missing results in a synthesis (arising from reporting biases). | Line159-160 |
| Certainty assessment | 15 | Describe any methods used to assess certainty (or confidence) in the body of evidence for an outcome. | Line140-147 |
| **RESULTS** | | |  |
| Study selection | 16a | Describe the results of the search and selection process, from the number of records identified in the search to the number of studies included in the review, ideally using a flow diagram. | Line191-198 |
|  | 16b | Cite studies that might appear to meet the inclusion criteria, but which were excluded, and explain why they were excluded. | Line201-202 |
| Study characteristics | 17 | Cite each included study and present its characteristics. | Line205-206 |
| Risk of bias in studies | 18 | Present assessments of risk of bias for each included study. | Line211-215 |
| Results of individual studies | 19 | For all outcomes, present, for each study: (a) summary statistics for each group (where appropriate) and (b) an effect estimate and its precision (e.g. confidence/credible interval), ideally using structured tables or plots. | Line217-263 |
| Results of syntheses | 20a | For each synthesis, briefly summarise the characteristics and risk of bias among contributing studies. | Line217-263 |
|  | 20b | Present results of all statistical syntheses conducted. If meta-analysis was done, present for each the summary estimate and its precision (e.g. confidence/credible interval) and measures of statistical heterogeneity. If comparing groups, describe the direction of the effect. | Line217-263 |
|  | 20c | Present results of all investigations of possible causes of heterogeneity among study results. | Line217-263 |
|  | 20d | Present results of all sensitivity analyses conducted to assess the robustness of the synthesized results. | Line266-271 |
| Reporting biases | 21 | Present assessments of risk of bias due to missing results (arising from reporting biases) for each synthesis assessed. | Line211-215 |
| Certainty of evidence | 22 | Present assessments of certainty (or confidence) in the body of evidence for each outcome assessed. | Line274-286 |
| **DISCUSSION** | | |  |
| Discussion | 23a | Provide a general interpretation of the results in the context of other evidence. | Line299-309 |
|  | 23b | Discuss any limitations of the evidence included in the review. | Line402-412 |
|  | 23c | Discuss any limitations of the review processes used. | Line402-412 |
|  | 23d | Discuss implications of the results for practice, policy, and future research. | Line387-401 |
| **OTHER INFORMATION** | | |  |
| Registration and protocol | 24a | Provide registration information for the review, including register name and registration number, or state that the review was not registered. | Not Applicable |
|  | 24b | Indicate where the review protocol can be accessed, or state that a protocol was not prepared. | Not Applicable |
|  | 24c | Describe and explain any amendments to information provided at registration or in the protocol. | Not Applicable |
| Support | 25 | Describe sources of financial or non-financial support for the review, and the role of the funders or sponsors in the review. | Line432-440 |
| Competing interests | 26 | Declare any competing interests of review authors. | Line422-430 |
| Availability of data, code and other materials | 27 | Report which of the following are publicly available and where they can be found: template data collection forms; data extracted from included studies; data used for all analyses; analytic code; any other materials used in the review. | Line443-444 |

Supplementary Table S2. Search terms employed in the literature search.

|  | PubMed |
| --- | --- |
|  | (Fatty Acids, Omega-3[MeSH Terms]) OR (N-3 Fatty Acid[MeSH Terms])) OR (Acid, N-3 Fatty[MeSH Terms])) OR (Fatty Acid, N-3[MeSH Terms])) OR (N 3 Fatty Acid[MeSH Terms])) OR (Omega-3 Fatty Acid[MeSH Terms])) OR (Acid, Omega-3 Fatty[MeSH Terms])) OR (Fatty Acid, Omega-3[MeSH Terms])) OR (Omega 3 Fatty Acid[MeSH Terms])) OR (Omega-3 Fatty Acids[MeSH Terms])) OR (n-3 Oil[MeSH Terms])) OR (n 3 Oil[MeSH Terms])) OR (Oil, n-3[MeSH Terms])) OR (n3 Oil[MeSH Terms])) OR (Oil, n3[MeSH Terms])) OR (n-3 Fatty Acids[MeSH Terms])) OR (n 3 Fatty Acids[MeSH Terms])) OR (n-3 Polyunsaturated Fatty Acid[MeSH Terms])) OR (n 3 Polyunsaturated Fatty Acid[MeSH Terms])) OR (n-3 PUFA[MeSH Terms])) OR (n 3 PUFA[MeSH Terms])) OR (n 3 PUFA[MeSH Terms])) OR (n3 Fatty Acid[MeSH Terms])) OR (Fatty Acid, n3[MeSH Terms])) OR (n3 PUFA[MeSH Terms])) OR (PUFA, n3[MeSH Terms])) OR (n3 Polyunsaturated Fatty Acid[MeSH Terms])) OR (n3 Oils[MeSH Terms])) OR (Omega 3 Fatty Acids[MeSH Terms])) OR (n-3 Oils[MeSH Terms])) OR (n 3 Oils[MeSH Terms])) OR (antioxidant)) AND (Coronary Artery Disease[MeSH Terms]) OR (Artery Disease, Coronary[Title/Abstract])) OR (Artery Diseases, Coronary[Title/Abstract])) OR (Coronary Artery Diseases[Title/Abstract])) OR (Coronary Arteriosclerosis[Title/Abstract])) OR (Arterioscleroses, Coronary[Title/Abstract])) OR (Coronary Arterioscleroses[Title/Abstract])) OR (Arteriosclerosis, Coronary[Title/Abstract])) OR (Atherosclerosis, Coronary[Title/Abstract])) OR (Atheroscleroses, Coronary[Title/Abstract])) OR (Coronary Atheroscleroses[Title/Abstract])) OR (Coronary Atherosclerosis[Title/Abstract])) OR (Coronary Atherosclerosis[Title/Abstract])) OR (Left Main Coronary Disease[Title/Abstract])) OR (Left Main Disease[Title/Abstract])) OR (Left Main Diseases[Title/Abstract])) |
|  | Web of science |
| #1 | TS=(Coronary Artery Disease OR CAD OR Artery Disease, Coronary OR Artery Diseases, Coronary OR Coronary Artery Diseases OR Coronary Arteriosclerosis OR Arterioscleroses, Coronary OR Coronary Arterioscleroses OR Arteriosclerosis, Coronary OR Atherosclerosis, Coronary OR Atheroscleroses, Coronary OR Coronary Atheroscleroses OR Coronary Atherosclerosis OR Left Main Coronary Artery Disease OR Left Main Coronary Disease OR Left Main Disease OR Left Main Diseases ) |
| #2 | TS=(Fatty Acids, Omega-3 OR Ω-3 OR N-3 Fatty Acid OR Acid, N-3 Fatty OR Fatty Acid, N-3 OR N 3 Fatty Acid OR Omega-3 Fatty Acid OR Acid, Omega-3 Fatty OR Fatty Acid, Omega-3 OR Omega 3 Fatty Acid OR Omega-3 Fatty Acids OR n-3 Oil OR n 3 Oil OR Oil, n-3 OR n3 Oil OR Oil, n3 OR n-3 Fatty Acids OR n 3 Fatty Acids OR n-3 Polyunsaturated Fatty Acid OR n 3 Polyunsaturated Fatty Acid OR n-3 PUFA OR n 3 PUFA OR PUFA, n-3 OR n3 Fatty Acid OR Fatty Acid, n3 OR n3 PUFA OR PUFA, n3 OR n3 Polyunsaturated Fatty Acid OR n3 Oils OR Omega 3 Fatty Acids OR n-3 Oils OR n 3 Oils OR antioxidant ) |
| #3 | **#1 AND #2** |
|  | Cochrane |
| #1 | (Omega-3 Fatty Acid):ab,ti,kw OR (Acid, Omega-3 Fatty):ab,ti,kw OR (Fatty Acid, Omega-3):ab,ti,kw OR (Omega 3 Fatty Acid):ab,ti,kw OR (Omega-3 Fatty Acids):ab,ti,kw OR (n-3 Oil):ab,ti,kw OR (Oil, n-3):ab,ti,kw OR (n 3 Oil):ab,ti,kw OR (n3 Oil):ab,ti,kw OR (Oil, n3):ab,ti,kw OR (n-3 Fatty Acids):ab,ti,kw OR (n 3 Fatty Acids):ab,ti,kw OR (Omega 3 Fatty Acids):ab,ti,kw OR (n-3 PUFA):ab,ti,kw OR (PUFA, n-3):ab,ti,kw OR (n 3 PUFA):ab,ti,kw OR (n3 Fatty Acid):ab,ti,kw OR (Fatty Acid, n3):ab,ti,kw OR (n3 PUFA):ab,ti,kw OR (PUFA, n3):ab,ti,kw OR (n3 Polyunsaturated Fatty Acid):ab,ti,kw OR (n3 Oils):ab,ti,kw OR (n-3 Oils):ab,ti,kw OR (n 3 Oils):ab,ti,kw OR (N-3 Fatty Acid):ab,ti,kw OR (Acid, N-3 Fatty):ab,ti,kw OR (Fatty Acid, N-3):ab,ti,kw OR (N 3 Fatty Acid):ab,ti,kw OR (n-3 Polyunsaturated Fatty Acid):ab,ti,kw OR (n 3 Polyunsaturated Fatty Acid):ab,ti,kw OR (antioxidant) |
| #2 | (Coronary Artery Disease):ab,ti,kw OR (CAD):ab,ti,kw OR (Artery Disease, Coronary):ab,ti,kw OR (Artery Diseases, Coronary):ab,ti,kw OR (Coronary Artery Diseases):ab,ti,kw OR (Coronary Arteriosclerosis):ab,ti,kw OR (Arterioscleroses, Coronary):ab,ti,kw OR (Coronary Arterioscleroses):ab,ti,kw OR (Arteriosclerosis, Coronary):ab,ti,kw OR (Atherosclerosis, Coronary):ab,ti,kw OR (Atheroscleroses, Coronary):ab,ti,kw OR (Coronary Atheroscleroses):ab,ti,kw OR (Coronary Atherosclerosis):ab,ti,kw OR (Left Main Coronary Artery Disease):ab,ti,kw OR (Left Main Coronary Disease):ab,ti,kw OR (Left Main Disease):ab,ti,kw OR (Left Main Diseases) |
| #3 | **#1 AND #2** |
|  | Embase |
| #1 | 'fatty acids, omega-3':ab,ti OR 'ω-3':ab,ti OR 'n-3 fatty acid':ab,ti OR 'acid, n-3 fatty':ab,ti OR 'fatty acid, n-3':ab,ti OR 'n 3 fatty acid':ab,ti OR 'omega-3 fatty acid':ab,ti OR 'acid, omega-3 fatty':ab,ti OR 'fatty acid, omega-3':ab,ti OR 'omega 3 fatty acid':ab,ti OR 'omega-3 fatty acids':ab,ti OR 'n-3 oil':ab,ti OR 'n 3 oil':ab,ti OR 'oil, n-3':ab,ti OR 'n3 oil':ab,ti OR 'oil, n3':ab,ti OR 'n-3 fatty acids':ab,ti OR 'n 3 fatty acids':ab,ti OR 'n-3 polyunsaturated fatty acid':ab,ti OR 'n 3 polyunsaturated fatty acid':ab,ti OR 'n-3 pufa':ab,ti OR 'n 3 pufa':ab,ti OR 'pufa, n-3':ab,ti OR 'n3 fatty acid':ab,ti OR 'fatty acid, n3':ab,ti OR 'n3 pufa':ab,ti OR 'pufa, n3':ab,ti OR 'n3 polyunsaturated fatty acid':ab,ti OR 'n3 oils':ab,ti OR 'omega 3 fatty acids':ab,ti OR 'n-3 oils':ab,ti OR 'n 3 oils':ab,ti OR 'antioxidant':ab,ti |
| #2 | 'Coronary Artery Disease ':ab,ti OR 'CAD ':ab,ti OR 'Artery Disease, Coronary ':ab,ti OR 'Artery Diseases, Coronary ':ab,ti OR 'Coronary Artery Diseases ':ab,ti OR 'Coronary Arteriosclerosis ':ab,ti OR 'Arterioscleroses, Coronary ':ab,ti OR 'Coronary Arterioscleroses ':ab,ti OR 'Arteriosclerosis, Coronary ':ab,ti OR 'Atherosclerosis, Coronary ':ab,ti OR 'Atheroscleroses, Coronary ':ab,ti OR 'Coronary Atheroscleroses ':ab,ti OR 'Coronary Atherosclerosis ':ab,ti OR 'Left Main Coronary Artery Disease ':ab,ti OR 'Left Main Coronary Disease ':ab,ti OR 'Left Main Disease ':ab,ti OR 'Left Main Diseases ':ab,ti |
| #3 | **#1 AND #2 AND** |

Supplementary Table S3. Calculation of the raw proportion of agreement by Cohen’s Kappa statistic.

| Categories | Author 1 | Author 2 |
| --- | --- | --- |
|  | Studies (5537) | Studies (5537) |
| Title-abstract screening | | |
| Total items coded idividually | 5537 | |
| Screening decision—include | 806 | 934 |
| Both judges agree to include | 726 | 726 |
| Both judges agree to exclude | 4523 | 4523 |
| Only the reviewer wants to include | 80 | 208 |
| Cohen’s kappa (% of agreement) | 0.804 | |
| Full-text screening | | |
| Total items coded individually | 208 | |
| Screening decision—include | 25 | 23 |
| Both judges agree to include | 22 | 22 |
| Both judges agree to exclude | 180 | 180 |
| Only the reviewer wants to include | 3 | 1 |
| Cohen’s kappa (% of agreement) | 0.859 | |

Supplemental Table S4: Reason for exclusion of retrieved articles

| NO. | Article title | Publication year | Reason for exclusion | Refs. |
| --- | --- | --- | --- | --- |
| 1 | Effect of low W-6/W-3 fatty acid ratio Paleolithic style diet in patients with acute coronary syndromes: A randomized, single blind, controlled trial. | 2012 | Without sufficient data | [1] |
| 2 | Omega-3 polyunsaturated fatty acids increase plasma adiponectin to leptin ratio in stable coronary artery disease. | 2013 | Without sufficient data | [2] |
| 3 | A combined effect of Cavacurcumin, Eicosapentaenoic acid (Omega-3s), Astaxanthin and Gamma–linoleic acid (Omega-6)(CEAG) in healthy volunteers-a randomized, double-blind, placebo-controlled study. | 2020 | Stuides in general population | [3] |
| 4 | Eicosapentaenoic acid (EPA) from highly concentrated n− 3 fatty acid ethyl esters is incorporated into advanced atherosclerotic plaques and higher plaque EPA is associated with decreased plaque inflammation and increased stability. | 2010 | No relevant outcome reported | [4] |
| 5 | Omega-3 polyunsaturated fatty acid supplementation improves lipid metabolism and endothelial function by providing a beneficial eicosanoid-pattern in patients with acute myocardial infarction: A randomized, controlled trial. | 2021 | No relevant outcome reported | [5] |
| 6 | The effects of rice bran oil on left ventricular systolic function, cardiometabolic risk factors and inflammatory mediators in men with coronary artery disease: a randomized clinical trial. | 2021 | rice bran oil intervention | [6] |
| 7 | Omega-3 fatty acids in high-risk cardiovascular patients: a meta-analysis of randomized controlled trials. | 2010 | No relevant outcome reported | [7] |
| 8 | Omega-3 polyunsaturated fatty acids and cardiovascular diseases. | 2009 | No relevant outcome reported | [8] |
| 9 | Омега-3 полиненасыщенные жирные кислоты у больных с гипертриглицеридемиями и атеросклерозом. | 2021 | non-English | [9] |
| 10 | The effect of n− 3 fatty acids on coronary atherosclerosis: Results from SCIMO, an angiographic study, background and implications. | 2001 | Without sufficient data | [10] |

Table S5.Subgroup analysis of outcomes by time, dose, type, country, mean age and disease state.

|  | Number of studies | SMD | 95% CI | Heterogeneity between studies | P Value | Tau2 |
| --- | --- | --- | --- | --- | --- | --- |
| **TG** | | | | | | |
| **Time** |  |  |  |  |  |  |
| ≤6 monthes | 11 | -0.30 | -0.54: -0.06 | I^2^=58.5 | 0.007 | 0.0921 |
| >6 monthes | 11 | -0.25 | -0.35: -0.07 | I^2^=8.5 | 0.363 | 0.0047 |
| **Dose** |  |  |  |  |  |  |
| ≤2g/day | 17 | -0.22 | -0.39: -0.06 | I^2^=47.4 | 0.016 | 0.052 |
| >2g/day | 5 | -0.31 | -0.54: -0.09 | I^2^=17 | 0.306 | 0.0118 |
| **Type** |  |  |  |  |  |  |
| EPA | 8 | -0.26 | -0.55: 0.027 | I^2^=68.8 | 0.002 | 0.1123 |
| EPA+DHA | 11 | -0.24 | -0.395: -0.088 | I^2^=16.5 | 0.287 | 0.0108 |
| Others | 3 | -0.27 | -0.635: 0.089 | I^2^=0 | 0.561 | 0 |
| **Country** |  |  |  |  |  |  |
| Europe | 5 | -0.20 | -0.36: -0.03 | I^2^=0 | 0.415 | 0 |
| Asia | 15 | -0.25 | -0.43: -0.06 | I^2^=50.2 | 0.014 | 0.0641 |
| North America | 2 | -0.54 | -0.92: -0.16 | I^2^=0 | 0.344 | 0 |
| **Mean age** |  |  |  |  |  |  |
| <60 | 8 | -0.30 | -0.92: -0.16 | I^2^=0 | 0.651 | 0 |
| ≥60 | 13 | -0.22 | -0.42: -0.02 | I^2^=59.6 | 0.003 | 0.0744 |
| NA | 1 | -0.16 | -0.78: 0.46 | - | - | 0 |
| **Disease state** |  |  |  |  |  |  |
| CAD | 19 | -0.27 | -0.41: -0.12 | I^2^=44.7 | 0.019 | 0.0431 |
| ACS | 3 | -0.09 | -0.40: 0.22 | I^2^=0 | 0.377 | 0 |
|  |  |  |  |  |  |  |
| **TC** | | | | | | |
| **Time** |  |  |  |  |  |  |
| ≤6 monthes | 11 | -0.25 | -0.47: -0.03 | I^2^=52.4 | 0.021 | 0.0717 |
| >6 monthes | 10 | 0.02 | -0.12: 0.17 | I^2^=0 | 0.973 | 0 |
| **Dose** |  |  |  |  |  |  |
| ≤2g/day | 17 | -0.15 | -0.31: 0.01 | I^2^=46.1 | 0.020 | 0.0493 |
| >2g/day | 4 | -0.01 | -0.29: 0.26 | I^2^=0 | 0.682 | 0 |
| **Type** |  |  |  |  |  |  |
| EPA | 8 | -0.29 | -0.55: -0.03 | I^2^=62.5 | 0.009 | 0.0847 |
| EPA+DHA | 10 | -0.06 | -0.22: 0.09 | I^2^=0 | 0.792 | 0 |
| Others | 3 | 0.255 | -0.11: 0.62 | I^2^=0 | 0.908 | 0 |
| **Country** |  |  |  |  |  |  |
| Europe | 4 | 0.04 | -0.17: 0.25 | I^2^=0 | 0.515 | 0 |
| Asia | 15 | -0.18 | -0.36: -0.01 | I^2^=45.9 | 0.027 | 0.0539 |
| North America | 2 | -0.11 | -0.48: 0.26 | I^2^=0 | 0.438 | 0 |
| **Mean age** |  |  |  |  |  |  |
| <60 | 12 | -0.19 | -0.40: 0.01 | I^2^=53.0 | 0.015 | 0.0648 |
| ≥60 | 8 | -0.05 | -0.22: 0.12 | I^2^=0 | 0.703 | 0 |
| NA | 1 | 0.35 | -0.27: 0.98 | - | - | 0 |
| **Disease state** |  |  |  |  |  |  |
| CAD | 18 | -0.14 | -0.30: 0.01 | I^2^=44.1 | 0.024 | 0.0462 |
| ACS | 3 | -0.04 | -0.35: 0.27 | I^2^=0 | 0.546 | 0 |
|  |  |  |  |  |  |  |
| **HDL-C** | | | | | | |
| **Time** |  |  |  |  |  |  |
| ≤6 monthes | 10 | -0.02 | -0.19: 0.15 | I^2^=14.6 | 0.308 | 0.0106 |
| >6 monthes | 10 | 0.16 | 0.02: 0.31 | I^2^=0 | 0.968 | 0 |
| **Dose** |  |  |  |  |  |  |
| ≤2g/day | 16 | 0.06 | -0.06: 0.17 | I^2^=0 | 0.584 | 0 |
| >2g/day | 4 | 0.19 | -0.09: 0.46 | I^2^=0 | 0.488 | 0 |
| **Type** |  |  |  |  |  |  |
| EPA | 8 | 0.06 | -0.09: 0.21 | I^2^=0 | 0.700 | 0 |
| EPA+DHA | 9 | 0.06 | -0.10: 0.22 | I^2^=4.4 | 0.399 | 0.0029 |
| Others | 3 | 0.28 | -0.09: 0.64 | I^2^=4.1 | 0.353 | 0.0044 |
| **Country** |  |  |  |  |  |  |
| Europe | 4 | 0.14 | -0.07: 0.36 | I^2^=0 | 0.595 | 0 |
| Asia | 14 | 0.09 | -0.03: 0.22 | I^2^=0 | 0.833 | 0 |
| North America | 2 | -0.29 | -0.88: 0.30 | I^2^=59.3 | 0.117 | 0.1073 |
| **Mean age** |  |  |  |  |  |  |
| <60 | 12 | 0.05 | -0.08: 0.18 | I^2^=0 | 0.983 | 0 |
| ≥60 | 7 | 0.09 | -0.18: 0.37 | I^2^=51.1 | 0.056 | 0.0678 |
| NA | 1 | 0.25 | - | - | - | 0 |
| **Disease state** |  |  |  |  |  |  |
| CAD | 17 | 0.07 | -0.04: 0.18 | I^2^=0 | 0.590 | 0 |
| ACS | 3 | 0.11 | -0.22: 0.44 | I^2^=10.4 | 0.328 | 0.0092 |
|  |  |  |  |  |  |  |
| **LDL-C** | | | | | | |
| **Time** |  |  |  |  |  |  |
| ≤6 monthes | 9 | -0.23 | -0.47: 0.01 | I^2^=53.2 | 0.029 | 0.0701 |
| >6 monthes | 11 | 0.02 | -0.13: 0.16 | I^2^=14.5 | 0.306 | 0.0084 |
| **Dose** |  |  |  |  |  |  |
| ≤2g/day | 15 | -0.17 | -0.35: 0.00 | I^2^=51.1 | 0.012 | 0.0577 |
| >2g/day | 5 | 0.06 | -0.15: 0.28 | I^2^=14 | 0.325 | 0.0092 |
| **Type** |  |  |  |  |  |  |
| EPA | 8 | -0.29 | -0.51: -0.07 | I^2^=46.4 | 0.071 | 0.0439 |
| EPA+DHA | 9 | -0.02 | -0.21: 0.17 | I^2^=36.6 | 0.126 | 0.0294 |
| Others | 3 | 0.29 | -0.07: 0.65 | I^2^=0 | 0.880 | 0 |
| **Country** |  |  |  |  |  |  |
| Europe | 4 | 0.13 | -0.09: 0.35 | I^2^=2.3 | 0.381 | 0.0013 |
| Asia | 14 | -0.21 | -0.39: -0.03 | I^2^=44.2 | 0.038 | 0.0491 |
| North America | 2 | 0.08 | -0.36: 0.52 | I^2^=59.2 | 0.118 | 0.0635 |
| **Mean age** |  |  |  |  |  |  |
| <60 | 13 | -0.16 | -0.33: 0.01 | I^2^=45.7 | 0.036 | 0.0421 |
| ≥60 | 6 | -0.01 | -0.31: 0.28 | I^2^=52.1 | 0.063 | 0.0698 |
| NA | 1 | 0.17 | -0.45: 0.79 | - | - | 0 |
| **Disease state** |  |  |  |  |  |  |
| CAD | 17 | -0.11 | -0.28: 0.05 | I^2^=54.0 | 0.004 | 0.0587 |
| ACS | 3 | -0.06 | -0.37: 0.24 | I^2^=0 | 0.649 | 0 |
|  |  |  |  |  |  |  |
| **Plaque** | | | | | | |
| **Time** |  |  |  |  |  |  |
| ≤6 monthes | 1 | -0.12 | -0.64: 0.39 | - | - | 0 |
| >6 monthes | 6 | -0.10 | -0.27: 0.06 | I^2^=5.7 | 0.308 | 0.0026 |
| **Dose** |  |  |  |  |  |  |
| ≤2g/day | 3 | -0.09 | -0.32: 0.14 | I^2^=0 | 0.872 | 0 |
| >2g/day | 4 | -0.16 | -0.45: 0.12 | I^2^=40.4 | 0.169 | 0.0338 |
| **Type** |  |  |  |  |  |  |
| EPA | 3 | -0.11 | -0.33: 0.11 | I^2^=0 | 0.98 | 0 |
| EPA+DHA | 4 | -0.16 | -0.47: 0.15 | I^2^=43 | 0.154 | 0.0436 |
| **Country** |  |  |  |  |  |  |
| Europe | 2 | 0.03 | -0.20: 0.26 | I^2^=0 | 0.669 | 0 |
| Asia | 5 | -0.19 | -0.39: 0.01 | I^2^=0 | 0.428 | 0 |
| **Mean age** |  |  |  |  |  |  |
| <60 | 1 | -0.06 | -0.54: 0.41 | - | - | 0 |
| ≥60 | 6 | -0.11 | -0.27: 0.06 | I^2^=5.5 | 0.382 | 0.0025 |
|  |  |  |  |  |  |  |

SMD: standardized mean difference; CI: confidence intervals; EPA: eicosapentaenoic acid; DHA: docosahexaenoic acid; TG: triglycerides; TC: total cholesterol; HDL-C: high-density lipoprotein ; LDL-C: low-density lipoprotein.

Supplementary Table S6. Results of Egger's Regression Test and Begg's Rank Correlation Test for various outcome measures.

|  | Egger's test | Begg's Test |
| --- | --- | --- |
| TG | *P*=0.846 | *P*=0.499 |
| HDL-C | *P*=0.851 | *P*=0.347 |
| TC | *P*=0.857 | *P*=0.319 |
| LDL-C | *P*=0.530 | *P*=0.770 |
| Plaque | *P*=0.205 | *P*=0.548 |

TG: triglycerides; TC: total cholesterol; HDL-C: high-density lipoprotein; LDL-C: low-density lipoprotein.

Supplementary Table S7. GRADE evidence table for the effects of omega-3 fatty acids on on blood lipids and plaques in patients with coronary atherosclerotic heart.

| **Certainty assessment** | | | | | | | **№ of patients** | | **Effect** | | **Certainty** | **Importance** |
| --- | --- | --- | --- | --- | --- | --- | --- | --- | --- | --- | --- | --- |
| **№ of studies** | **Study design** | **Risk of bias** | **Inconsistency** | **Indirectness** | **Imprecision** | **Other considerations** | **[intervention]** | **[comparison]** | **Relative (95% CI)** | **Absolute (95% CI)** |  |  |
| **Plaque** | | | | | | | | | | | | |
| 6 | randomised trials | serious^1^ | no serious inconsistency | serious^2^ | no serious inconsistency | none | 371 | 323 | - | SMD 0.1 lower (0.257 lower to 0.504 higher) | ⨁⨁◯◯ Low | IMPORTANT |
| **TG** | | | | | | | | | | | | |
| 22 | randomised trials | Serious^1^ | no serious risk of bias | no serious inconsistency | no serious inconsistency | none | 888 | 797 | **-** | SMD 0.25 lower (0.38 to 0.11 lower) | ⨁⨁⨁◯ Moderate | IMPORTANT |
| **HDL-C** | | | | | | | | | | | | |
| 18 | randomised trials | Serious^1^ | no serious inconsistency | no serious indirectness | no serious inconsistency | none | 745 | 679 | - | SMD 0.08 higher (0.03 lower to 0.18 higher) | ⨁⨁⨁◯ Moderate | IMPORTANT |
| **LDL-C** | | | | | | | | | | | | |
| 18 | randomised trials | no serious inconsistency | no serious inconsistency | no serious indirectness | Serious^3^ | none | 844 | 754 | - | SMD 0.1 lower (0.25 lower to 0.04 higher) | ⨁⨁⨁◯ Moderate | IMPORTANT |
| **TC** | | | | | | | | | | | | |
| 19 | randomised trials | no serious inconsistency | no serious inconsistency | no serious indirectness | Serious^3^ | none | 766 | 700 | - | SMD 0.12 lower (0.234 to 0.02 lower) | ⨁⨁⨁◯ Moderate | IMPORTANT |

GRADE：Grading of Recommendations Assessment, Development, and Evaluation; CI: confidence interval; SMD: standardized mean difference; TG: triglycerides; HDL-C: high-density lipoprotein ; LDL-C: low-density lipoprotein; TC: total cholesterol.

#### Explanations

^1^. There is a risk of bias. Downgraded.

^2^. Serious indirectness since only six studies were available. Downgraded.

^3^. There is a Imprecision. Downgraded.


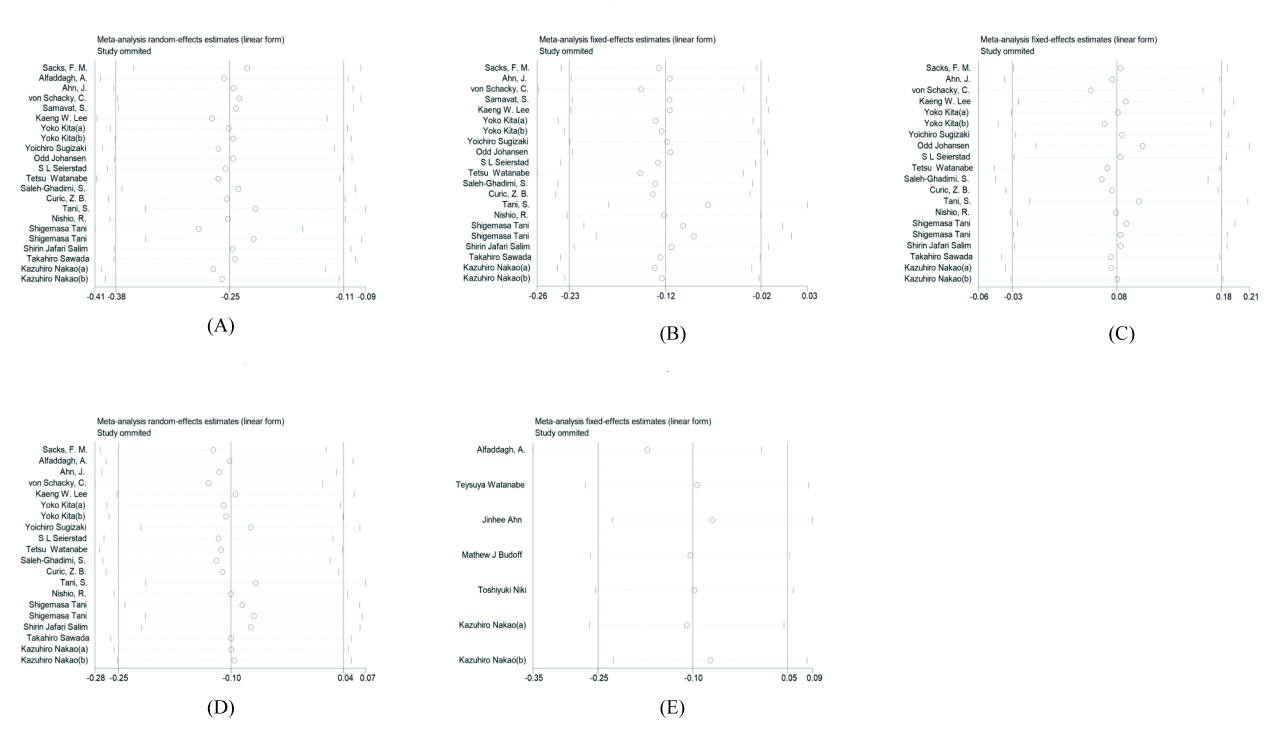


Fig. S1. Sensitivity analysis results of different outcome indicators. (A ) triglyceride (TG); (B) total cholesterol (TC); (C) high density lipoprotein cholesterol(HDL-C); (D) low-density lipoprotein cholesterol(LDL-C); (E) Plaque volume.


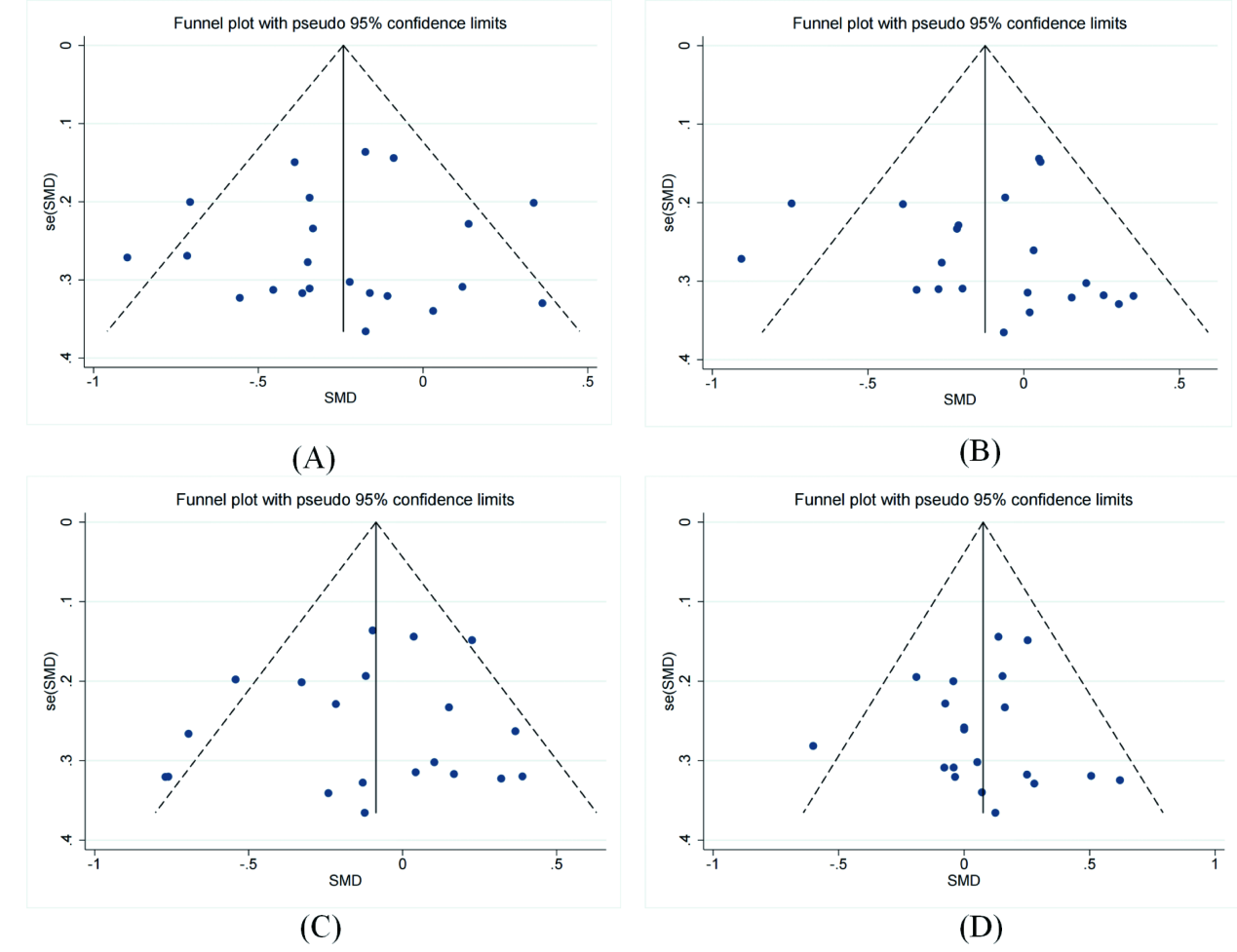


Fig. S2. Funnel plots for different outcome indicators. (A ) triglyceride(TG); (B) total cholesterol (TC); (C) high density lipoprotein cholesterol(HDL-C); (D) low-density lipoprotein cholesterol(LDL-C); SMD：standardised mean difference；CI: confidence interval.

**Reference**

[1] R. Singh, J. Fedacko, V. Vargova, et al., Effect of low W-6/W-3 fatty acid ratio Paleolithic style diet in patients with acute coronary syndromes: A randomized, single blind, controlled trial, 4 (2012) 71.

[2] M. Mostowik, G. Gajos, J. Zalewski, et al., Omega-3 polyunsaturated fatty acids increase plasma adiponectin to leptin ratio in stable coronary artery disease, Cardiovasc. Drugs Ther. 27 (2013) 289–295.

[3] D. Birudaraju, L. Cherukuri, A. Kinninger, et al., A combined effect of Cavacurcumin, Eicosapentaenoic acid (Omega-3s), Astaxanthin and Gamma–linoleic acid (Omega-6) (CEAG) in healthy volunteers- a randomized, double-blind, placebo-controlled study, Clin. Nutr. ESPEN 35 (2020) 174–179.

[4] A.L. Cawood, R. Ding, F.L. Napper, et al., Eicosapentaenoic acid (EPA) from highly concentrated n−3 fatty acid ethyl esters is incorporated into advanced atherosclerotic plaques and higher plaque EPA is associated with decreased plaque inflammation and increased stability, Atherosclerosis 212 (2010) 252–259.

[5] M. Yuan, Y. Zhang, T. Hua, et al., Omega-3 polyunsaturated fatty acid supplementation improves lipid metabolism and endothelial function by providing a beneficial eicosanoid-pattern in patients with acute myocardial infarction: A randomized, controlled trial, Clin. Nutr. 40 (2021) 445–459.

[6] M. Mahdavi-Roshan, A. Salari, Z. Ghorbani, et al., The effects of rice bran oil on left ventricular systolic function, cardiometabolic risk factors and inflammatory mediators in men with coronary artery disease: A randomized clinical trial, Food Funct. 12 (2021) 4446–4457.

[7] K.B. Filion, F. El Khoury, M. Bielinski, et al., Omega-3 fatty acids in high-risk cardiovascular patients: A meta-analysis of randomized controlled trials, BMC Cardiovasc. Disord. 10 (2010), 24.

[8] C.J. Lavie, R.V. Milani, M.R. Mehra, et al., Omega-3 polyunsaturated fatty acids and cardiovascular diseases, J. Am. Coll. Cardiol. 54 (2009) 585–594.

[9] А.J.К. Сусеков, Омега-3 полиненасыщенные жирные кислоты у больных с гипертриглицеридемиями и атеросклерозом, 61 (2021) 88-96.

[10] C. von Schacky, K. Baumann, P. Angerer, The effect of n-3 fatty acids on coronary atherosclerosis: Results from SCIMO, an angiographic study, background and implications, Lipids 36 (2001) S99–102.
